# Microbial Ecology at the Nexus of Food Safety and Biotechnology With Ecological Mechanisms, Risks, and Emerging Innovations

**DOI:** 10.1155/ijfo/6618960

**Published:** 2026-06-23

**Authors:** Jackline A. Tepson, Daniel S. Agyirifo

**Affiliations:** ^1^ Department of Molecular Biology and Biotechnology, University of Cape Coast, Cape Coast, Ghana, ucc.edu.gh

**Keywords:** biopreservation, fermentation, food microbiome, food safety, metagenomics, microbial ecology, omics, probiotics, synthetic biology

## Abstract

Food systems are complex microbial ecosystems in which microorganisms play dual and often contrasting roles as agents of foodborne contamination and as essential drivers of food production and biotechnological innovation. Microbial ecology provides an integrative framework for understanding how microbial interactions, environmental conditions, and human interventions shape food safety outcomes and technological processes. This narrative integrative review is aimed at synthesizing current literature on microbial ecology at the nexus of food safety and food biotechnology and at identifying key research gaps and future directions. In this study, peer‐reviewed journal articles addressing microbial interactions, contamination pathways, and ecological mechanisms relevant to food safety and biotechnology published between 2015 and 2025 were retrieved from major scientific databases and were synthesized using a narrative integrative approach. The review highlights ecological factors including microbial competition, stress adaptation, and biofilm formation across pre‐ and postharvest environments. At the same time, these same ecological principles are harnessed in food biotechnology to drive controlled fermentations, enhance shelf life through biopreservation, develop functional probiotics and enzymes, and engineer microbial systems via synthetic biology. Advances in high‐throughput sequencing technologies, including whole genome sequencing, metagenomics, and multiomics integration, are identified as transformative tools for linking food‐associated microbial community structure to functional outcomes. Despite significant progress, challenges remain in translating ecological insights into reliable industrial and regulatory practices due to microbial complexity, data integration limitations, and safety considerations. The review positions microbial ecology as a strategic framework for advancing food safety, biotechnological innovation, and sustainable food systems.

## 1. Introduction

Food safety is the process of handling, preparing, and storing food in ways that prevent foodborne illness. Food can become contaminated at any point during slaughtering or harvesting, processing, storage, distribution, transportation, and preparation [1]. Food systems are complex ecosystems riddled with abundant microbial life, ranging from organisms that are pathogenic to those that are beneficial. On one hand, microbial activity can lead to undesirable outcomes such as food spoilage and the proliferation of foodborne pathogens, posing significant risks to public health and inflicting substantial economic burdens on the food industry [2]. An estimated 600 million, almost 1 in 10 people in the world, fall ill after eating contaminated food, and 420,000 die every year [3, 4]. Each year, $110 billion is lost in productivity and medical expenses resulting from unsafe food in low‐ and middle‐income countries [5]. While microorganisms are often associated with foodborne illness, they are indispensable to food production, particularly through fermentation processes that enhance flavor and texture and extend shelf life [6]. Furthermore, the inherent capabilities of microorganisms are increasingly being harnessed through biotechnological applications to develop novel food ingredients, processing aids, and innovative preservation strategies [7]. Conventional food safety detection methods often depend on culture‐based approaches, though these fail to fully represent the complexity and dynamics of microbial ecosystems [8]. Recent advancements in microbial ecology offer insights into contamination pathways, resilience mechanisms, and biotechnological applications, presenting opportunities for sustainable solutions in food systems [9, 10].

Microbial ecology is the discipline that examines the interactions among microorganisms, their environments, and external interventions, providing a framework for understanding how microbial communities function, adapt, and influence both contamination dynamics and beneficial processes within food systems, as illustrated by studies on microbial community assembly and interaction networks in diverse habitats [11–13]. By understanding these dynamics, innovative solutions that go beyond simple eradication to prevent contamination and promote ecological balance can be developed. Biotechnological applications derived from microbial ecological research hold transformative potential for food safety. Applications such as biopreservation, probiotics, fermentation, microbial biosensors, and synthetic biology leverage ecological principles to enhance food quality, extend shelf life, and improve safety [14]. Traditional fermentation, the linchpin of food preservation and cultural heritage, is being utilized to harness the metabolic activities of microorganisms like lactic acid bacteria (LAB) and yeasts [7, 15]. Modern biotechnology builds upon this knowledge to develop defined starter cultures and create novel fermented products with enhanced sensory and nutritional profiles [16]. The field of functional foods leverages specific microbes (probiotics) or substrates that modulate microbial activity (prebiotics) to promote gut health [17]. Biopreservation utilizes beneficial microorganisms or their metabolites to inhibit the growth of undesirable microbes, offering natural alternatives to chemical preservatives [18]. More recently, synthetic biology is opening new frontiers, enabling the engineering of microorganisms to produce valuable food ingredients, enhance nutritional content, or even create entirely new food sources in a sustainable manner [19]. These advancements also support sustainability by reducing reliance on chemical preservatives and antibiotics [20].

Despite the growing interest in the field of microbial ecology, there are gaps in understanding microbial community dynamics and their practical applications [21]. Existing literature often lacks a comprehensive synthesis of findings relevant to both food safety and biotechnological innovations. The intricate relationship between microbial contamination and biotechnological innovation underscores the central importance of microbial ecology in modern food science. This review is aimed at synthesizing current literature on the role of microbial ecology in understanding and mitigating microbial contamination and in driving biotechnological innovations that improve food safety and also at identifying research gaps and future directions.

## 2. Methodology

This study was conducted as a narrative integrative review aimed at synthesizing current knowledge on the role of microbial ecology in food safety and biotechnological applications. A structured but nonsystematic approach was adopted to capture both foundational concepts and recent technological advancements in the field.

### 2.1. Search Strategy

A comprehensive literature search was performed using PubMed, Scopus, Web of Science, and Google Scholar databases. Publications from 2015 to 2025 were prioritized to ensure relevance to recent developments, although seminal earlier works were included where necessary for conceptual grounding. Search terms included combinations of *microbial ecology*, *food safety*, *food microbiome*, *fermentation*, *biopreservation*, *probiotics*, *metagenomics*, *synthetic biology*, and *food biotechnology*. Boolean operators (“AND” and “OR”) were applied to refine search results.

### 2.2. Study Selection and Eligibility

Studies were selected based on relevance to microbial interactions within food systems and their implications for contamination control or biotechnological innovation.

#### 2.2.1. Inclusion Criteria

• Peer‐reviewed journal articles published in English.
• Studies addressing microbial ecology in food production, processing, preservation, or fermentation.
• Research linking microbial community dynamics to food safety, quality, or biotechnology.
• In addition to original research articles, selected review articles were included to provide conceptual background, support thematic synthesis, and ensure comprehensive coverage of the topic.

#### 2.2.2. Exclusion Criteria

• Non–peer‐reviewed literature (conference abstracts, editorials, and opinion pieces).
• Studies focused solely on clinical or environmental microbiology without food relevance.
• Articles lacking ecological or functional interpretation.

Titles and abstracts were initially screened for relevance, followed by full‐text evaluation. A total of 178 articles met the inclusion criteria and were retained for inclusion in the final synthesis.

### 2.3. Data Extraction and Synthesis

Key information extracted from selected studies included microbial taxa involved, ecological interactions, environmental drivers, methodological approaches, and practical implications for food safety or biotechnology. Findings were synthesized thematically across major domains, including contamination pathways, fermentation ecology, biopreservation, enzyme biotechnology, probiotics, and emerging omics‐based tools. Rather than quantitative meta‐analysis, this review emphasizes conceptual integration and ecological interpretation of evidence.

### 2.4. Synthesis of Evidence

The following section synthesizes published evidence on microbial ecology in food systems, focusing on contamination pathways, ecological drivers of microbial persistence, and applications in food biotechnology.

### 2.5. Microbial Ecology and Food Safety

Microbial contamination in food systems arises from complex ecological interactions across production, processing, and distribution environments. Rather than isolated events, contamination reflects the ability of pathogens to persist, adapt, and compete within food‐associated microbial communities.

### 2.6. Sources and Pathways of Contamination

**Figure 1:**
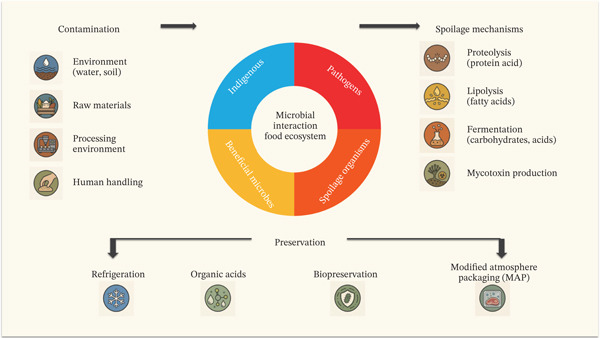
Conceptual overview of microbial ecology in food systems illustrating contamination sources (environment, raw materials, processing, and human handling), microbial interactions (pathogens, spoilage organisms, and beneficial microbes), ecological processes (competition, biofilm formation, and fermentation), and preservation strategies (biopreservation, organic acids, refrigeration, and modified atmosphere packaging). This figure is a modified illustration based on an initial AI‐generated image using ChatGPT. The authors substantially revised and refined the figure, including all structural elements, labels, and conceptual relationships, to reflect the framework presented in this study.

Microbial contamination in food is of highest concern for humans as it affects public health, safety, and the global economy as a whole. Contamination originates from water, soil, and processing environments, introducing pathogens like *Salmonella* and *Listeria* [22] (Figure 1). Understanding the ecological origins and transmission routes of foodborne pathogens is important for effective control and prevention. Food contamination occurs at any point along the food chain [23]. Pathogens can be introduced into food during primary production, particularly for fresh produce, of which sources include contaminated soil (which can be a natural reservoir for pathogens like *Listeria monocytogenes*), improperly treated animal manure used as fertilizer, contaminated irrigation water (especially surface water), and fecal shedding by wild or domestic animals [24]. Insects such as flies can also serve as pathways for contamination by acting as vectors, transferring pathogens from contaminated sources to food produce [25, 26]. Food can also be contaminated in food processing facilities, of which sources include raw materials, personnel, and the environment [1]. Biofilms on equipment surfaces enhance microbial resilience, complicating decontamination efforts [27]. Major bacterial, viral, and parasitic pathogens implicated in foodborne disease, their sources, ecological niches, and control strategies are summarized in Tables 1 and 2. These pathogens can lead to severe gastrointestinal diseases, with some strains causing life‐threatening complications.

**Table 1:** Most common pathogens implicated foodborne outbreaks, sources, and control measures.

Pathogen	Primary food sources	Common symptoms	Key control measures	References
*Salmonella*	Raw meat, poultry, eggs, unpasteurized dairy, produce	Diarrhea, fever, abdominal cramps, vomiting	Cook thoroughly, avoid cross‐contamination, refrigerate promptly	[28]
*Campylobacter*	Raw poultry, unpasteurized milk, contaminated water	Diarrhea (often bloody), cramps, fever, vomiting	Cook poultry thoroughly, avoid cross‐contamination, use pasteurized milk	[29]
*E. coli* (STEC)	Undercooked ground beef, unpasteurized milk/juice, produce	Severe abdominal cramps, diarrhea (often bloody), vomiting	Cook ground beef thoroughly, wash produce, avoid unpasteurized products	[30, 31]
*Listeria monocytogenes*	Unpasteurized dairy, ready‐to‐eat meats, soft cheeses, produce	Fever, muscle aches, nausea, diarrhea; can be severe	Avoid high‐risk foods if at risk, cook thoroughly, practice good hygiene	[32]
*Clostridium botulinum*	Improperly canned foods, honey (infants)	Double vision, blurred vision, slurred speech, paralysis	Proper canning techniques, avoid giving honey to infants	[33]
*Staphylococcus aureus*	Cooked foods left at room temperature; foods handled by infected persons, beef, eggs	Nausea, vomiting, abdominal cramps, diarrhea (rapid onset)	Practice good hygiene, refrigerate cooked foods promptly	[34, 35]
*Bacillus cereus*	Rice, sauces, raw milk, soups left at room temperature	Diarrheal: diarrhea and cramps; emetic: nausea and vomiting	Refrigerate leftovers promptly, keep hot foods hot, cool foods quickly	[36, 37]
*Norovirus*	Contaminated food or water, surfaces, raw oysters, produce	Nausea, vomiting, diarrhea, abdominal cramps	Practice good hygiene, wash hands thoroughly, cook shellfish thoroughly	[38, 39]

**Table 2:** Ecological niches and safety concerns of key foodborne pathogens.

Pathogen	Niches	Survival mechanism	Associated illness	References
*Listeria monocytogenes*	Ready‐to‐eat (RTE) foods, dairy products, fresh produce, soil	Psychrotrophic, biofilm formation, acid/salt tolerance, intracellular survival	Listeriosis	[40, 41]
*Salmonella* spp.	Poultry, eggs, meat, fresh produce, contaminated water/feed	Biofilm formation, survival in diverse environments (soil, water), resistance to drying, gut colonization	Salmonellosis	[42]
*Escherichia coli* O157:H7 and other STEC	Undercooked ground beef, fresh produce, contaminated water, cattle feces	Acid tolerance, biofilm formation, low infectious dose, toxin production	Hemorrhagic colitis, HUS	[43]
*Campylobacter* spp.	Poultry, raw milk, contaminated water	Microaerophilic, motile, gut colonization (esp. poultry), sensitive to drying/heat	Campylobacteriosis	[44]
*Staphylococcus aureus*	Human skin/nasal passages, dairy products (esp. raw milk), meat	Toxin production (heat‐stable enterotoxins), salt tolerance, biofilm formation, potential MRSA strains	Staphylococcal food poisoning	[45]
*Bacillus cereus*	Soil, rice, starchy foods, dairy products	(heat resistant), toxin production (emetic/diarrheal), psychrotrophic strains exist	Emetic/diarrheal illness	[46]

### 2.7. Ecological Factors Influencing Pathogen Survival and Proliferation

The survival and proliferation of pathogens in food systems are significantly influenced by various ecological factors [47]. In preharvest environments, pathogen dynamics are affected by soil characteristics, including type, moisture, pH, and organic matter [48]. Additionally, irrigation practices and prevailing weather conditions, such as temperature, humidity, and UV radiation, play critical roles in the survival of pathogens [49]. For example, cool, moist conditions often favor bacterial survival. The physiology of the plant, including its surface characteristics and growth stage, also impacts pathogen dynamics. Indigenous microbial communities, such as those found in the phyllosphere and rhizosphere, can either inhibit or facilitate pathogen growth through competitive interactions [50, 51]. Pathogens can attach to produce surfaces and may even internalize into plant tissues via natural openings or wounds, which protects them from surface decontamination methods [52]. In postharvest and processing environments, factors such as temperature, water activity, pH, and nutrient availability affect pathogen growth or survival [53]. Pathogens also form biofilms that provide an additional layer of protection, enhancing pathogen persistence on surfaces and their resistance to environmental stresses [54]. Pathogens require organic compounds, carbohydrates, and proteins for their survival and growth. In environments where beneficial microbes are suppressed, the availability of nutrients can enhance pathogen survival, highlighting the importance of maintaining a balanced microbial community [55]. Beneficial microbes can produce antimicrobial substances that inhibit pathogen growth, creating a competitive exclusion environment. Host factors, such as the presence of animals or humans, further influence pathogen dynamics. Many pathogens are zoonotic and can be carried asymptomatically by animals, leading to contamination of food products [43]. Human behaviors, including improper food handling and inadequate cooking, can create favorable conditions for pathogen proliferation, prompting the need for education on safe food practices [3]. Moreover, environmental stressors, including antimicrobial agents, also play a role in pathogen survival. While preservatives can inhibit growth, some pathogens may develop resistance, necessitating ongoing monitoring and adaptation of food safety practices [56] (Table 2).

### 2.8. Microbial Ecology as a Driver of Food Biotechnology

Beyond contamination control, microbial ecology provides the foundation for food biotechnology by enabling the rational use of microbial interactions to enhance food quality, functionality, and safety. While foodborne microbes pose hazards, many microbes are harnessed as productive “biotools.” Microbial ecology insight has enabled breakthroughs in fermentation, enzyme technology, probiotics, and biopreservation.

### 2.9. Fermentation Ecology: Controlled Microbial Ecosystems

Fermentation is the process of utilizing the metabolic activities of microorganisms such as acids or alcohol production to produce various food products with enhanced shelf life, flavor, and nutritional value [57]. Microbial communities originate from the natural (indigenous) microbiota present on raw materials (plants, milk, and meat) or in the processing environment, through the practice of back‐slopping (using a portion of a previous successful fermentation to inoculate a new batch), or via the deliberate addition of selected starter cultures [58]. Fermentations involve dynamic microbial consortia where the relative abundance and activity of different microbial groups change over time in response to evolving environmental conditions (e.g., decreasing pH, nutrient depletion, and accumulation of metabolites) [59]. This process of microbial succession is governed by ecological principles like competition for substrates, tolerance to environmental stresses (e.g., acidity and salt), and metabolic interactions (e.g., cross‐feeding and inhibition). Metabolic flux within fermentation consortia shapes the quality of the end product. Microorganisms compete for limiting nutrients (such as amino acids, nitrogen, peptides, or vitamins), and this competition channels metabolic pathways in distinct ways. For example, heterogeneous communities in spontaneous fermentations may divert nitrogen into a broader array of amino acid–derived volatiles, whereas defined starter cultures often channel flux toward uniform acid and ester production. Recent multiomics studies show that modulating nutrient availability can redirect metabolic flux and alter flavor outcomes. In one case, adding the amino acid L‐leucine to a yeast starter markedly increased flux through the leucine catabolism pathway, yielding higher levels of isoamyl alcohol and isoamyl acetate (key flavors in beer and sake) [60]. Quantitative tools like genome‐scale metabolic modeling and flux analysis are increasingly used to connect these nutrient competitions with specific flavor compound profiles, helping explain why spontaneous and inoculated fermentations produce different sensory outcomes. Different types of fermentation are characterized by distinct dominant microbial groups (Table 3). Lactic acid fermentation is dominated by LAB, including genera like *Lactobacillus*, *Lactiplantibacillus*, *Lacticaseibacillus*, *Leuconostoc*, *Streptococcus*, *Lactococcus*, *Pediococcus*, and *Weissella*. LAB convert sugars primarily to lactic acid (homofermentative) or to ethanol/acetic acid and CO_2_ (heterofermentative) [61]. This type of fermentation is mostly used in the production of yogurt, cheese, sauerkraut, kimchi, some fermented sausages, and sourdough [62]. Alcoholic fermentation is primarily driven by yeasts, especially *Saccharomyces cerevisiae*, converting sugars to ethanol and CO_2_ [63]. This is used mostly in the process of brewing (beer), winemaking, and leavening bread (often in conjunction with LAB in sourdough). Mold fermentation utilizes *Aspergillus* spp. (e.g., soy sauce, miso, and sake—often for enzyme production), *Rhizopus* spp. (e.g., tempeh), and *Penicillium* spp. (e.g., cheese ripening—Roquefort and Camembert) [64]. Acetic acid fermentation utilizes acetic acid bacteria (AAB), such as *Acetobacter* and *Gluconobacter*, to oxidize ethanol to acetic acid, and it is mostly used in vinegar production [65]. Understanding fermentation as a process of managed ecological succession, where conditions are set to favor the desired microbial pathways while suppressing undesirable ones (spoilage and pathogens) through mechanisms like acidification and antimicrobial production, is key to controlling and optimizing these processes. Furthermore, the vast microbial diversity harbored within traditional, often geographically localized, fermented foods represents a significant, largely untapped resource for discovering novel strains with unique metabolic capabilities applicable to food biotechnology. Modern biotechnology advances have emerged as an innovative tool that can be used to tap into this space. Some of these advances include controlled starter cultures and defined microbial consortia. Metagenomic studies also now guide the design of starter communities with desired traits [66]. In addition, new fermented products (kombucha, kefir, and fermented plant proteins) are developed for health benefits [67]. Synthetic biology is even being used to engineer strains to produce novel aromas or nutraceuticals, turning waste substrates into value‐added foods [68] (Table 4).

**Table 3:** Dominant microbial taxa in various fermented food categories.

Fermented food category	Primary microbial groups	Dominant genera	Primary functional role(s)	References
Yogurt/fermented milks	Thermophilic LAB	*Streptococcus thermophilus*, *Lactobacillus delbrueckii* subsp. *bulgaricuss*	Rapid acidification, texture (EPS), flavor (acetaldehyde)	[69]
Cheese (starter‐dominated)	Mesophilic/thermophilic LAB	*Lactococcus lactis*, *Streptococcus thermophilus*, *Lactobacillus* spp.	Acidification, proteolysis (early ripening), flavor precursors	[70]
Cheese (ripened, e.g., blue and mold)	LAB, yeasts, molds	*Lactococcus*, *Lactobacillus*, *Debaryomyces*, *Geotrichum*, *Penicillium roqueforti/camemberti*	Acidification, proteolysis, lipolysis, flavor/aroma development, texture modification	[70, 71]
Sauerkraut/kimchi	Hetero/homofermentative LAB	*Leuconostoc mesenteroides* (early), *Lactiplantibacillus plantarum*, *Lactobacillus* spp., *Weissella* spp.	Acidification (lactic, acetic), gas (CO_2_), flavor, texture, pathogen inhibition	[72]
Sourdough	LAB (hetero/homo), yeasts	*Fructilactobacillus sanfranciscensis*, *Lactiplantibacillus*, *Levilactobacillus*, *Saccharomyces cerevisiae*, *Kazachstania* spp.	Acidification, leavening (CO_2_), flavor/aroma, proteolysis, phytate hydrolysis	[70]
Fermented sausage	LAB, coagulase‐neg. staphylococci (CNS), yeasts	*Lactobacillus sakei/curvatus*, *Pediococcus* spp., *Staphylococcus xylosus/carnosus*, *Debaryomyces hansenii*	Acidification, proteolysis, lipolysis, color development (nitrate reduction by CNS), flavor/aroma	[69]
Soy sauce/miso	Molds, yeasts, LAB	*Aspergillus oryzae/sojae* (koji stage), *Zygosaccharomyces rouxii*, *Tetragenococcus halophilus* (brine stage)	Enzyme production (proteases, amylases), flavor/aroma (salt‐tolerant fermentation)	[73]
Tempeh	Molds	*Rhizopus oligosporus*/*oryzae*	Mycelial growth binding beans, enzyme production (proteases, lipases), nutrient release	[74]
Beer/wine	Yeasts (primarily), sometimes LAB	*Saccharomyces cerevisiae/pastorianus*, *Brettanomyces* spp. (some styles), *Oenococcus oeni* (wine—malolactic)	Ethanol and CO_2_ production, flavor/aroma compounds (esters, phenols), malic acid	[73]

**Table 4:** Exploration of microbial ecology in food systems.

Food category/system	Microbial ecology focus	Methodology	Location	Key findings	Applications/implications	References
Dairy: cheese and plant	Facility and cheese microbiome analysis	Shotgun metagenomic, MAG reconstruction	Austria, Spain, Ireland, and Italy	Identified highly complex microbial communities within cheese and processing plants. Facility‐specific microbial signatures were uncovered, with genes linked to flavor and probiotic properties. MAGs revealed the presence of antibiotic resistance genes.	Highlights traceability in facility‐specific microbes, aiding quality assurance and safety management. Provides a base for improving flavor and probiotic activity while addressing AMR concerns.	[75]
Meat: processing facilities	Microbiome shaping by treatments and conditions	Third‐generation sequencing	United States	Showed antimicrobial treatments and temperature selection shape the microbiota in meat plants. Persistent pathogens like *E. coli* O157:H7 were identified. Detected facility‐wide microbiome resilience despite interventions.	Data aids in optimizing sanitation protocols and targeted antimicrobials. Sequencing insights allow proactive pathogen monitoring/surveillance and reduction strategies.	[76]
Beverage: Chinese rice wine	Microbiota vs. fermentation quality; spoilage	16S rRNA and ITS sequencing (Illumina), plus metagenomics (shotgun)	China (Shaoxing)	The abundance of *Lactobacillus* correlated with final wine quality. High abundance of *Lb. brevis* early on was linked to spoilage. Shotgun sequencing revealed *Lb. brevis* growth triggered off‐flavors. Functional gene analysis highlighted the roles of malolactic fermentation and biotin synthesis.	Demonstrates that microbial monitoring can predict wine quality or spoilage conditions. Useful for controlling fermentation quality in rice wine breweries.	[77]
Dairy: cheddar cheese	Community composition in artisanal vs. industrial cheeses	16S rRNA amplicon sequencing; metabolomics (GC‐MS/LC‐MS)	Australia	Industrial and artisanal cheddars showed highly distinct bacterial communities. 16S profiling found just 16 OTUs (mainly *Streptococcus*, *Lactococcus*, *Lactobacillus*) that made up > 70% of each cheese’s microbiome. Artisanal cheeses had additional genera (e.g., *Staphylococcus*).	Integrative microbiome–metabolome analysis can link microbes to flavor compounds. Understanding core genera informs starter selection and quality control in cheese production.	[78]
Fermented foods (diverse)	Community profiling; AMR and health‐related genes	Shotgun metagenomics (58 fermented foods)	Various (global artisanal samples)	Fermented foods clustered by substrate (dairy, sugar, brine). Dairy‐based ferments had the lowest microbial diversity. Fermented foods were enriched for “health‐associated” functions (e.g., vitamin/cofactor pathways) and carbohydrate degradation genes. Many MAGs (127 high‐quality) were recovered.	Mapping of AMR and functional genes across ferments can guide safety and product development. Results suggest that fermentation can enhance health‐linked metabolites, guiding probiotic/biotech exploitation.	[79]
Dairy: fermented milks (cheeses, kefir, ayran, etc.)	Taxonomic/functional profiling; AMR gene survey	Shotgun metagenomics (Illumina NovaSeq, MAG reconstruction)	Russia (multiple regions)	Artisanal Russian fermented dairy was dominated by lactic acid bacteria (*Lb. delbrueckii*, *St. thermophilus*, etc.) with species varying by product type (e.g., *Lb. helveticus* in cottage cheese). MAG analysis found hundreds of biosynthetic gene clusters (bacteriocins, polyketide synthases) suggesting antimicrobial potential. ABR gene counts were generally low (e.g., none in ayran and 47 in one cheese).	Highlights novel probiotic candidates (LAB with useful metabolisms like GABA and vitamins) and shows that most strains lack worrisome AMR. Data can aid the selection of beneficial cultures and ensure safety by monitoring ABR gene transfer potential.	[80]
Meat: fresh beef (ground and nonground)	Baseline microbiota; potential spoilage taxa	16S rRNA amplicon (Illumina MiSeq); functional prediction	South Korea	The spoilage genera (e.g., lactic acid bacteria and *Pseudomonas* spp.) were more prevalent in summer (July). Predicted functions indicated fermentation of glucose.	Knowledge of core and spoilage microbes informs shelf life management. Seasonal shifts (e.g., higher spoilage bacteria in summer) suggest targeted interventions (e.g., chilled transport) to reduce spoilage and extend shelf life of beef.	[81]
Meat: fresh pork (processing plant)	Microbiome on meat vs. equipment; line/time differences	16S rRNA amplicon (Illumina; custom workflow)	United States (pork plant, two processing lines)	Processing design and sanitation shape distinct microbial communities on meat and surfaces.	Identifies critical control points to implement interventions to improve food safety and quality in pork production.	[82]
Beverage: kombucha (fermented tea)	Community composition; metabolic potential	Shotgun metagenomics	China	Kombucha microbiomes were dominated by acetic acid bacteria (*Komagataeibacter*, *Gluconacetobacter*, *Gluconobacter*) with some yeast (*Kluyveromyces*). Functional analysis showed abundant carbohydrate‐active enzymes (glycosyltransferases, glycoside hydrolases) and pathways for vitamin and amino acid metabolism. Taxonomic profiles varied by sample.	Detailed community and function profiles can aid in standardizing kombucha production for flavor/health. For example, identifying key microbes and enzymes can help optimize fermentation conditions or assess the probiotic potential of kombucha.	[83]
Fermented: pickled vegetables (kimchi, etc.)	Community composition; ARGs and probiotics	Shotgun metagenomics (Illumina)	Saudi Arabia (market samples)	Common LAB included *Levilactobacillus namurensis*, *Lactiplantibacillus pentosus*, *Weissella confusa*, etc. Four putative novel species were identified. Remarkably, 285 antibiotic resistance genes (covering ~20 antibiotic classes) were detected (mainly on Enterobacteriaceae contigs).	Highlights both beneficial strains (potential probiotics) and safety concerns (high ARG load). Suggests need for improved hygiene (to limit *Enterobacteriaceae*) in fermented vegetable production. Also offers new isolates for biotech (e.g., unique lactobacilli).	[84]
Seafood: European sea bass (whole vs. fillet)	Spoilage community dynamics under storage	16S rRNA high‐throughput sequencing	Greece	Modified atmosphere packaging (MAP) shifted the community: for example, at day middle of storage, *Carnobacterium*/*Shewanella* emerged, and at the end of life, *Serratia* dominated in MAP at 12°C.	Guides the seafood industry on storage practices. Informs shelf life predictions and targeted antimicrobials.	[85]
Beverage: traditional rice‐based alcoholic drinks	Community profiling of ethnic beverages	16S rRNA amplicon (Illumina MiSeq)	India (Assam)	Indigenous rice beers from five ethnic tribes were all Firmicutes‐rich. Genera like *Pediococcus*, *Lactobacillus*, *Bacillus*, *Leuconostoc*, and *Acetobacter*, as well as some *Erwinia/Klebsiella*, were found at high abundance across samples. The microbial composition varied by tribal recipe.	Cataloging the microbiome of traditional foods can link microbial functions (e.g., probiotic lactobacilli) to health benefits celebrated by local cultures. Can guide the development of starter cultures that preserve cultural heritage and enhance nutrition.	[86]
Fermented: Mao (“hairy”) tofu (fermented soybean)	Core microbiome (fungi and bacteria)	High‐throughput sequencing (ITS, LSU, 16S)	China (Yunnan)	Sequencing of Mao tofu (outside rind vs. inner core) found ~170 bacterial genera enriched mainly near surface biofilms. Functional profiling aiding better validations.	Suggests optimal biofilm combinations for healthiness and shelf life while improving ecofermentation via waste or scale tool guidance for future decisions.	[87]

### 2.10. Microbial Enzymes in Food Processing

Microbial enzymes have become indispensable tools in the modern food industry, offering a diverse range of applications from baking to brewing and from dairy production to fruit juice clarification [88]. Microorganisms are a rich source of enzymes that are used extensively in food processing to improve efficiency, yield, texture, flavor, and nutritional value [89]. These microbial enzymes, derived from various bacteria and fungi, are being utilized in the food industry to improve the quality and characteristics of food products [88]. Some of these enzymes include amylases, proteases, lipases, cellulases, and pectinases, and each enzyme performs a specific function during food processing. Amylases are derived from *Bacillus subtilis* and *Aspergillus oryzae*, and their main function is to hydrolyze starch into simpler sugars [90]. In baking, amylases are used to improve dough texture and fermentation, resulting in bread with enhanced quality [91]. Similarly, they are used in brewing to convert starches into fermentable sugars, which are essential for alcohol production [92]. Proteases are derived from organisms like *Bacillus licheniformis* and *Rhizopus oryzae*, and their function is to break down proteins into peptides and amino acids, influencing both the flavor and texture of food [93]. In cheese production, proteases contribute to curd formation and the development of unique flavor profiles [94]. In meat processing, proteases are used to tenderize meat, making it more palatable [95]. Lipases are sourced from bacteria like *Pseudomonas* spp. and fungi like *Candida* spp., and they catalyze the hydrolysis of fats and oils [96]. Lipases are mostly used in dairy production to enhance the flavors of cheeses and improve the quality of milk products [97]. Cellulases are derived from fungi such as *Trichoderma reesei*, and they break down cellulose, a major component of the plant cell walls [98]. This is particularly useful during fruit juice production, where cellulases clarify juices by breaking down cell walls, improving yield and overall quality [99]. Pectinases, commonly derived from *Aspergillus niger*, degrade pectin, a polysaccharide found in plant cell walls [100]. These enzymes are invaluable in fruit juice and wine production, improving juice extraction and clarification, as well as enhancing the texture of jams and jellies [101].

Future research efforts are more focused on exploring new sources of microbial enzyme production and engineering enzymes with enhanced properties [102]. Genetic engineering is being utilized to modify microbial strains, producing enzymes with improved stability, activity, and specificity for targeted applications [103]. Metagenomic approaches are also being utilized to explore different microbial communities in diverse environments, with the aim of discovering novel enzymes that can be used in food processing [104] (Table 5).

**Table 5:** Recent global case studies on microbial enzyme discovery and engineering for food processing.

Country	Enzyme developed	Key findings	Application	References
Italy	Engineered industrial enzymes	Mutagenesis and protein engineering enhanced thermostability and catalytic rates.	Used in baking and brewing, improving fermentation and texture.	[103]
China	Novel cellulases from bamboo pulp waste	Metagenomic and 16S rRNA sequencing uncovered cellulase genes from *Cloacibacterium*, *Paludibacter*, *Exiguobacterium*, *Acetivibrio*, *Tolumonas*, and *Clostridium*.	Applied in fiber degradation, beverage clarification, and plant‐based food processing.	[105]
China	*β*‐Glucosidase mutants from *Oenococcus oeni*	Two site‐mutated enzymes showed ~2.8–3.2× higher activity and better thermal stability than wild type.	Applied in flavor enhancement of wines and fermented beverages.	[106]
Norway/United Kingdom	Marine protease cocktail	Metagenomic sequencing identified marine bone‐degrading enzymes; enzyme blend degraded bone proteins efficiently.	Used to upcycle animal and fish bone waste into food‐grade protein powders.	[107]
Norway	Novel protease from sludge microbiome	Functional screening found protease active up to 50°C with broad substrate range.	Used in soy sauce and protein hydrolysate production.	[108]
Iran	Engineered *α*‐amylase from rumen metagenome	Discovered thermostable and pH‐tolerant *α*‐amylase.	Used in starch hydrolysis, brewing, and sweetener production.	[109]
Egypt	Cellulase and xylanase from *Bacillus pumilus*	Optimized enzyme production from a novel strain; high yield under controlled conditions.	Used in agrofood waste bioconversion and fiber modification in food formulations.	[110]

### 2.11. Probiotic and Prebiotic Development in Food Biotechnology

The growing understanding of microbial ecology in food biotechnology has profoundly influenced the development of probiotics and prebiotics, two functional components essential for promoting gut health and overall well‐being [111]. These developments leverage the complex interactions within the gut microbiome to enhance digestion, immune function, and disease prevention. Probiotics represent a direct method of introducing beneficial microbes [112, 113]. Commonly utilized probiotic genera include various species such as *Lactobacillus* (now distributed among several genera), *Bifidobacterium*, the yeast *Saccharomyces boulardii*, and select strains of *Enterococcus*, *Streptococcus*, and *Bacillus* [114]. These organisms are often sourced from traditional fermented foods but are increasingly delivered as defined strains in supplements or fortified products [115]. The beneficial effects of probiotics stem from their ecological interactions within the host gut [116]. They can outcompete potential pathogens for essential nutrients and binding sites on the intestinal lining. Furthermore, many probiotics actively inhibit harmful microbes by producing substances like organic acids, hydrogen peroxide, or specific antimicrobial peptides known as bacteriocins [117]. Beyond direct antagonism, probiotics engage with the host by modulating the immune system, often strengthening gut barrier function and influencing immune cell activity and responses, such as enhancing IgA production [118]. Their metabolic activities also contribute benefits, including the production of vitamins and health‐promoting short‐chain fatty acids (SCFAs) [119]. Complementing probiotics are prebiotics, which are nondigestible substrates selectively utilized by beneficial host microorganisms [120]. Typically, these are dietary fibers like inulin, fructo‐oligosaccharides (FOSs), and galacto‐oligosaccharides (GOSs). Unlike probiotics, prebiotics do not introduce new microbes but rather act as targeted nourishment for existing beneficial populations, particularly bifidobacteria and lactobacilli [121]. This selective feeding stimulates their growth and activity, leading to increased production of SCFAs like butyrate, which is a primary energy source for colon cells and has wider systemic health benefits [122]. The combination of probiotics and prebiotics in a single product, known as synbiotics, is aimed at enhancing the survival and efficacy of the probiotic component within the gut environment [123]. The success of both probiotic and prebiotic interventions is fundamentally governed by ecological principles. Efficacy is highly dependent on the specific microbial strain(s) used, the dosage administered, and crucially, the host’s unique existing gut microbiome composition, diet, and overall health status [124]. This highlights the personalized nature of gut microbiome modulation. Research continues to explore these complex interactions, including the potential of next‐generation probiotics derived from a wider range of commensal gut bacteria beyond the traditional LAB and bifidobacteria [125]. In essence, probiotics and prebiotics exemplify how understanding microbial ecology allows for targeted strategies to positively influence the gut microbiome for improved host health.

### 2.12. Biopreservation: Microbial Ecology as a Food Safety Tool

Biopreservation utilizes the natural antagonistic interactions among microorganisms to control the growth of spoilage organisms and pathogens in food, offering a clean‐label alternative or complement to traditional chemical preservatives [126]. This eco‐friendly approach leverages microbial ecology principles to enhance food safety, extend shelf life, and maintain sensory quality.

A biopreservation method is protective cultures, typically LAB, which are deliberately introduced into food to inhibit undesirable microbes [127]. These beneficial microbes suppress spoilage and pathogenic organisms through competition for nutrients and space, as well as by producing antimicrobial metabolites such as organic acids, hydrogen peroxide, diacetyl, reuterin, and bacteriocins [128]. Protective cultures are carefully selected for their strong antagonistic activity against microbes like *Listeria monocytogenes*, *Clostridium* species, and spoilage yeasts and molds, while ensuring they do not adversely affect the food’s flavor or texture [129]. For example, specific LAB strains are applied in meat products to control *Listeria*, and *Propionibacterium freudenreichii* is used in cheese to inhibit mold growth [130]. Selection processes often involve screening numerous isolates from food environments to identify strains with optimal efficacy, safety, and technological suitability.

The use of bacteriocins is another biopreservation method utilized in food biotechnology. Bacteriocins are antimicrobial peptides ribosomally synthesized by bacteria, mainly LAB, that target closely related bacterial species [131]. Nisin, produced by *Lactococcus lactis*, is the most well‐known and commercially used bacteriocin, effective against Gram‐positive pathogens including *Listeria*, *Staphylococcus*, and spore‐forming *Clostridium* and *Bacillus* [132]. Other bacteriocins include pediocin, sakacins, and enterocins. These peptides can be applied either as purified or semipurified additives or generated in situ by protective cultures. Their specificity and natural proteinaceous nature, which allows digestion in the human gut, make them attractive natural preservatives [133]. However, bacteriocins face challenges such as a limited spectrum of activity (often ineffective against Gram‐negative bacteria unless combined with membrane‐permeabilizing agents) [134], potential for resistance development, and issues with stability and delivery in complex food matrices [135].

Another innovative biopreservation tool is bacteriophages (phages), viruses that specifically infect and lyse bacteria. Phage therapy is re‐emerging as a precise method to control bacterial pathogens such as *Listeria*, *Salmonella*, and *E. coli* O157:H7 in food and processing environments [136]. Phages offer high host specificity, targeting harmful bacteria without disturbing beneficial microbiota or altering food sensory qualities [137]. Several phage products have obtained regulatory approvals for direct food application, including meats, poultry, and fresh produce [138]. A typical example is GRAS in the United States which is used for direct phage product approval application. Phages are also useful for decontaminating food contact surfaces and disrupting bacterial biofilms [138].

In addition to bacteria‐targeting agents, antifungal compounds produced by certain fungi and yeasts play an important role in controlling spoilage molds and yeasts [139]. These include organic acids like propionic and acetic acid, cyclic dipeptides, and specific antifungal proteins, commonly applied in dairy products and baked goods to enhance shelf life and product quality [140]. Biopreservation strategies depend mostly on manipulating microbial interactions, especially competition and antagonism, to shape the food microbial ecosystem toward safety and stability. By exploiting these natural microbial dynamics, biopreservation provides an effective, sustainable, and consumer‐friendly approach to food preservation that aligns with current demands for clean‐label and minimally processed products.

### 2.13. Emerging Tools Shaping Food Microbial Ecology

Traditional culture‐based methods remain a reference standard for detecting pathogens and spoilage microbes [141]. These methods often lack the resolution needed to definitively link foodborne illness cases to specific sources, especially for common pathogens [142]. While reliable for culturable organisms, culture methods require a much longer time to identify an organism. Furthermore, the method can miss out on viable‐but‐non–culturable (VBNC) cells. In order to authenticate isolates, cultures are often “coupled with modern tools such as PCR, immunoassays, next‐generation sequencing (NGS), biosensors, and MALDI‐TOF MS” to reduce detection time and improve specificity [143]. The advent of high‐throughput sequencing has ushered in a new era for microbial food safety surveillance and outbreak investigation, leading to improve detection time [144] (Table 6).

**Table 6:** Advanced methodologies for studying food microbial ecology.

Methodology	Principle/data type	Key applications in food context	Strengths	Limitations/challenges	References
WGS	Full genome sequence of isolate	Pathogen tracking/surveillance, source attribution, AMR/virulence prediction, strain characterization, root cause analysis	Highest resolution for strain comparison, comprehensive genetic info	Requires culturable isolate, data analysis/storage demands	[142]
Metagenomics (amplicon)	Targeted gene sequencing (e.g., 16S rRNA and ITS) from total DNA	Community composition profiling (bacteria/fungi), diversity analysis, widely used for broad surveys	Cost‐effective, established analysis pipelines, good for taxonomic overview	Limited functional insight, PCR biases, lower taxonomic resolution (often genus level), does not detect viruses	[145]
Metagenomics (shotgun)	Random sequencing of all DNA in a sample	Community composition, functional potential (gene content), ARG detection, virus/phage detection, strain‐level analysis possible	Culture‐independent, provides functional insights, higher resolution than amplicon, detects broad range of organisms	High cost; complex bioinformatic analysis; host DNA interference (high host‐to‐microbial DNA ratios in animal‐derived foods such as meat, and dairy reduce microbial read depth and require host DNA depletion steps); difficulty detecting low‐abundance organisms	[146, 147]
Transcriptomics (meta‐)	Sequencing of RNA transcripts (gene expression)	Identifying active genes/pathways, understanding microbial responses to environment, linking genotype to phenotype	Reveals active processes, dynamic view of community function	RNA instability, complex data analysis, quantitation challenges	[148]
Proteomics	Analysis of protein complement	Identifying expressed proteins and enzymes, understanding functional roles, and posttranslational modifications	Direct measure of functional molecules	Technically challenging, complex data analysis, lower throughput than sequencing	[149]
Metabolomics	Analysis of small molecule metabolites	Metabolic fingerprinting, identifying flavor/aroma compounds, detecting toxins/spoilage markers, understanding metabolic pathways	Closest link to phenotype, direct measure of biochemical output	Compound identification challenges, dynamic range issues, sample preparation complexity	[150]
Multiomics integration	Combining data from multiple “omics” layers	Holistic system understanding, linking structure‐function‐phenotype, elucidating interactions, building predictive models	Comprehensive view, mechanistic insights	Data integration complexity, bioinformatics expertise required, high cost	[151]
Synthetic biology/ecology	Engineering microbes/constructing defined communities	Ingredient production, enhanced fermentation, studying interactions, designing functional consortia, bioprospecting	Rational design, controlled experimentation, potential for novel applications	Safety/regulatory considerations (engineered organisms), predicting complex interactions, scalability	[152]

### 2.14. NGS: Whole Genome Sequencing (WGS) and Metagenomics

The advent of NGS technologies has revolutionized the study of food microbial ecology by enabling culture‐independent analysis at unprecedented depth and scale [145]. WGS of isolated microorganisms provides the ultimate resolution for strain‐level characterization [153]. In food safety, WGS is transforming outbreak investigations by allowing precise tracking of pathogen transmission pathways, differentiating between closely related strains, identifying virulence and AMR determinants directly from the genome, and facilitating rapid source attribution [154, 155]. Regulatory agencies and public health laboratories are increasingly adopting WGS for routine surveillance of key foodborne pathogens [156].

Since not all microorganisms are culturable, metagenomics evolved to sequence the total DNA extracted from a sample or an environment. This allows for the characterization of entire microbial communities without the need for cultivation. Amplicon sequencing (metataxonomic), typically targeting the 16S rRNA gene for bacteria or the ITS region for fungi, is widely used to profile community composition and diversity in various food matrices and environments [157]. Shotgun metagenomics sequences all DNA present, providing insights not only into community composition but also into the collective functional potential (gene content) of the microbiome, including the detection of ARGs [158]. While powerful, metagenomics faces challenges, such as the difficulty in reliably detecting low‐abundance pathogens in complex food matrices without prior enrichment and the computational complexity of analyzing large datasets [159]. Databases like FoodMicrobionet are being developed to collate and standardize food‐related metataxonomic data, facilitating broader ecological comparisons [160].

### 2.15. Multiomics Integration

While genomics reveals the potential of a microbial community, understanding its actual behavior requires integrating multiple layers of biological information. Multiomics approaches combine genomics with other “omics” technologies such as transcriptomics (studying gene expression via RNA), proteomics (studying protein profiles), and metabolomics (studying small molecule metabolites) to provide a more holistic view of microbial systems [161]. Multiomics can elucidate the complex metabolic cross‐talk between different members of a microbial community and identify metabolic bottlenecks or burdens, particularly in engineered strains used for precision fermentation. By generating a functional blueprint of a food microbial ecosystem, multiomics moves beyond descriptive ecology toward a more predictive understanding, identifying key players and metabolic markers associated with desired quality attributes or potential safety hazards [162]. However, the effective integration and interpretation of large, multilayered datasets remain a significant bioinformatic challenge.

### 2.16. Synthetic Biology and Synthetic Ecology

Synthetic biology provides innovative tools for the rational design and engineering of microorganisms with novel or enhanced functionalities relevant to food production [163]. This includes engineering microbes to produce specific ingredients, improve fermentation efficiency, utilize alternative feedstocks (like CO_2_ or methanol), or enhance nutritional properties [163, 164]. Complementing this, synthetic ecology involves constructing simplified, defined microbial communities (synthetic consortia) from well‐characterized strains [165]. These model systems allow researchers to study fundamental ecological principles, such as interspecies interactions, community stability, and emergent properties, in a controlled and tractable manner, simplifying the complexity inherent in natural ecosystems. For instance, synthetic gut communities are used to investigate metabolic interactions and the effects of diet on microbial dynamics [166]. The knowledge gained from synthetic ecology can inform the design of robust and functional multispecies starter cultures for food fermentations or probiotic applications [167]. Combining the engineering capabilities of synthetic biology with the controlled testing environment of synthetic ecology offers a powerful approach for developing novel microbial consortia tailored for specific food applications, potentially enabling a form of “directed ecological evolution” toward desired community functions and stability [167].

## 3. Discussion

By integrating evidence across food production environments, fermentation systems, and biotechnological applications, this review highlights microbial ecology as a unifying framework connecting food safety challenges with innovation‐driven solutions.

### 3.1. Microbial Ecology as a Framework for Food Systems

Microbial ecology serves as a comprehensive framework for understanding the intricate interactions between microorganisms, food environments, and human interventions. The findings of this review show that microorganisms play a dual role within food systems, acting as both agents of contamination and beneficial microbes for food biotechnology and sustainability. This dual role places microbial ecology at the intersection of food safety and innovation, emphasizing that microorganisms operate as dynamic members of complex ecosystems whose interactions ultimately define food quality, safety, and functionality [168]. By applying core ecological principles, competition, succession, adaptation, and symbiosis, the food industry can move from reactive contamination control toward proactive ecosystem management that nurtures beneficial microbial processes while minimizing pathogenic risks.

### 3.2. Ecological Understanding of Foodborne Contamination

Viewing foodborne contamination through an ecological lens shows that pathogens persist not because of sporadic contamination events but due to their adaptive capacity to thrive within microbial networks. Pathogens such as *Listeria monocytogenes*, *Salmonella* spp., *Escherichia coli* O157:H7, and *Staphylococcus aureus* exhibit remarkable ecological plasticity, enabling survival across a wide range of environmental and processing conditions [41, 45]. Mechanisms such as quorum sensing, biofilm formation, and stress response systems contribute to their persistence in both food and environmental niches [45, 49]. Recent research indicates that *Listeria* can survive long term within mixed‐species biofilms, where interactions with LAB or *Pseudomonas* confer enhanced tolerance to disinfectants [169]. These findings suggest that controlling pathogens in the food system requires ecological interventions that will disrupt microbial networks and nutrient niches, rather than sole reliance on chemical sanitation. Strategies incorporating beneficial microbial consortia, phage biocontrol, and ecological modulation of processing environments represent a paradigm shift toward microbial community management for improved food safety.

### 3.3. Microbial Ecology and Food Biotechnology

Similarly, microbial ecology underpins the beneficial processes that define modern food biotechnology. Fermentation shows how ecological succession and association between microorganisms can be harnessed for food transformation. Mixed microbial consortia, comprising bacteria, yeasts, and sometimes molds, drive biochemical conversions that improve sensory, nutritional, and functional attributes of foods [15]. Advances in metagenomics, transcriptomics, and metabolomics have revealed that these microbial communities are not static but dynamically structured, with early colonizers modifying environmental conditions to favor successive species. For instance, in sourdough fermentations, heterofermentative *Lactobacillus* species lower the pH and generate organic acids that enable subsequent yeasts to dominate later stages, influencing flavor and aroma profiles [170]. Understanding such ecological dynamics facilitates rational starter culture design and the development of probiotic formulations with enhanced functional traits.

### 3.4. Biopreservation as an Ecological Strategy

The concept of biopreservation shows the application of ecological mechanisms to food safety enhancement. Protective cultures, particularly LAB, are key ecological agents that produce a variety of antimicrobial metabolites such as organic acids, hydrogen peroxide, and bacteriocins like nisin and pediocin [129]. These metabolites act through multiple mechanisms, membrane disruption, competitive exclusion, and nutrient depletion, to inhibit pathogenic and spoilage organisms. Similarly, bacteriophages offer host‐specific biocontrol of bacteria including *Listeria*, *Salmonella*, and *Campylobacter*, providing precision interventions without disturbing beneficial microbiota [138]. Emerging phage‐based products such as ListShield and SalmoFresh are now used in ready‐to‐eat foods, highlighting their commercial viability [171]. Yeasts and filamentous fungi also produce antifungal metabolites that protect dairy and bakery products, underscoring the diversity of ecological mechanisms available for sustainable preservation [172]. Collectively, these strategies align with consumer‐driven clean‐label initiatives by reducing chemical preservatives and promoting microbial balance.

### 3.5. Microbial Enzymes and Industrial Applications

Microbial enzymes further exemplify the industrial potential of microbial ecology. Enzymes such as proteases, amylases, lipases, and cellulases are central to food processing, texture modification, and flavor generation [88]. Recent metagenomic screening from extreme and underexplored environments, such as saline soils, compost, and fermented wastes, continues to uncover novel enzymes with improved thermostability and substrate specificity [104]. These enzymes support bioprocess efficiency while reducing environmental footprints, demonstrating how ecological diversity directly translates to biotechnological innovation. The integration of metagenomics and synthetic biology now enables enzyme optimization through gene editing and directed evolution, providing unprecedented control over enzyme performance for industrial applications.

### 3.6. Integration of Omics and Synthetic Biology

The emergence of high‐throughput sequencing, multiomics integration, and synthetic biology has transformed food microbial ecology from an observational field into a predictive science. WGS now enables source attribution, outbreak tracking, and the identification of virulence or antimicrobial resistance determinants with single‐nucleotide resolution [155]. Metagenomics and metabolomics reveal the structure and function of complex microbial communities, allowing researchers to map interspecies interactions and metabolic fluxes [145]. Moreover, synthetic biology extends these insights into practical applications by enabling the rational engineering of microbial consortia or individual strains for specific ecological outcomes, such as enhanced fermentation, bioactive compound production, and targeted pathogen inhibition [163]. These advancements mark a transition from reactive food safety measures toward proactive ecosystem engineering, where microbial behavior can be predicted and steered to achieve desired outcomes.

### 3.7. Challenges and Knowledge Gaps

Despite rapid technological progress, translating microbial ecological knowledge into reliable food safety and biotechnology solutions remains challenging. The complexity of multispecies interactions and spatial heterogeneity in biofilms complicates the prediction of microbial dynamics [21]. Many genes identified through metagenomics remain functionally uncharacterized, limiting the translation of genomic data into practical applications. Furthermore, the use of engineered or synthetic microorganisms in food raises ecological, ethical, and regulatory concerns that require harmonized safety assessments and international policy frameworks [173]. Regulatory approaches to engineered microbes differ between different jurisdictions. In the European Union, current law follows a precautionary model: The 2018 European Court of Justice decision ruled that organisms modified by newer techniques (e.g., CRISPR) are subject to existing GMO regulations. Thus, gene‐edited strains effectively face the same lengthy approval and labeling requirements as traditional transgenic GMOs. By contrast, the United States uses a product‐based framework. The USDA and FDA generally do not classify simple gene edits (without introduced foreign DNA) as GMOs. For example, CRISPR‐edited yeast or plant varieties with only small deletions have been exempted from regulation. As a result, most gene‐edited foods in the United States do not carry GMO labels unless their nutritional profile or allergen content has changed. These differences mean that industrial developers of synthetic biology solutions must navigate a patchwork: the EU’s strict GMO‐based regime versus the United States more lenient, trait‐focused regime [174, 175]. There is also a need for improved rapid, culture‐independent diagnostics capable of detecting low‐abundance pathogens and antimicrobial resistance genes in complex food matrices [176]. These challenges highlight the importance of cross‐disciplinary collaboration among microbiologists, bioinformaticians, and regulatory bodies to ensure safe and sustainable integration of microbial technologies.

### 3.8. Future Research Directions

Looking forward, the next decade of research should focus on deepening mechanistic understanding and expanding technological integration within microbial ecology. The combination of machine learning with multiomics data holds great promise for predictive modeling of microbial behavior, enabling early identification of contamination events and real‐time process optimization. Emerging biosensing technologies, such as CRISPR‐based and electrochemical sensors, can facilitate rapid and precise detection of pathogens and antimicrobial resistance markers directly in processing environments. Further exploration of microbial dark matter through metagenomic and single‐cell sequencing will likely reveal novel enzymes, secondary metabolites, and symbiotic interactions relevant to food safety and biotechnology. Advancements in synthetic biology should prioritize the design of minimal or synthetic microbial consortia capable of maintaining ecological stability in industrial fermentations. Moreover, future research must evaluate the ecological and evolutionary consequences of deploying engineered microorganisms and bacteriophages in food systems, ensuring that innovation aligns with ecological safety and regulatory responsibility. Integrating these emerging technologies within a system ecology framework will accelerate the transition toward intelligent, adaptive, and sustainable food ecosystems.

## 4. Conclusion

Microbial ecology has emerged as a unifying framework connecting food safety challenges with biotechnological innovation. By shifting focus from pathogen eradication to ecosystem management, ecological approaches enable more sustainable, resilient, and effective food safety strategies. Advances in fermentation science, biopreservation, probiotics, microbial enzymes, and synthetic biology demonstrate how beneficial microbial functions can be harnessed to improve food quality and public health. The integration of high‐throughput sequencing, multiomics, and synthetic ecology is transforming food microbiology into a predictive and design‐oriented discipline. However, addressing ecological complexity, regulatory concerns, and translational barriers remains essential for responsible implementation. Future research should prioritize interdisciplinary collaboration and ecosystem‐based management to ensure safe, innovative, and sustainable global food systems.

## Funding

No funding was received for this manuscript.

## Disclosure

All generated content was critically reviewed, edited, and verified by the authors to ensure accuracy and integrity such as Figure 1. After using these tools, the authors reviewed and edited the content as needed and take full responsibility for the content of the publication.

## Conflicts of Interest

The authors declare no conflicts of interest.

## References

[1] Marriott N. G. , Schilling M. W. , Gravani R. B. , Marriott N. G. , Schilling M. W. , and Gravani R. B. , Food Contamination Sources, Principles of Food Sanitation, 2018, Springer, 83–91, 10.1007/978-3-319-67166-6_5.

[2] Amubieya O. F. and Olawepo G. K. , Economic Consequences of Microorganisms in Food, Food Safety and Quality in the Global South, 2024, Springer, 533–560, 10.1007/978-981-97-2428-4_17.

[3] Mohammad A.-M. , Chowdhury T. , Biswas B. , and Absar N. , Food Poisoning and Intoxication: A Global Leading Concern for Human Health, Food Safety and Preservation, 2018, Elsevier, 307–352, 10.1016/B978-0-12-814956-0.00011-1.

[4] Oladunjoye A. O. and Awani-Aguma E. U. , Foodborne Illnesses: Prevention and Control, Food Safety and Toxicology: Present and Future Perspectives, 2023, Walter de Gruyter GmbH & Co KG, 10.1515/9783110748345-007.

[5] Unnevehr L. J. , Addressing Food Safety Challenges in Rapidly Developing Food Systems, Agricultural Economics. (2022) 53, no. 4, 529–539, 10.1111/agec.12724.

[6] Izah S. C. , Odubo T. C. , Ogwu M. C. , and Hait M. , Aspects of Microorganisms in the Food Industry, Food Safety and Quality in the Global South, 2024, Springer, 399–425, 10.1007/978-981-97-2428-4_13.

[7] Garba N. Y. , Fardami A. Y. , Adamu A. A. , Shehu A. A. , Ibrahim L. , Abdullahi S. , Aliyu A. , Abubakar A. S. , Salisu A. R. , and Abdulrahman M. , The Beneficial Roles of Microbes in Food Production and Preservation: A Review, International Journal of Science for Global Sustainability (IJSGS). (2025) 11, no. 1, 76–92, 10.57233/ijsgs.v11i1.784.

[8] Kabiraz M. P. , Majumdar P. R. , Mahmud M. M. C. , Bhowmik S. , and Ali A. , Conventional and Advanced Detection Techniques of Foodborne Pathogens: A Comprehensive Review, Heliyon. (2023) 9, no. 4, e15482, 10.1016/j.heliyon.2023.e15482, 37151686. 37151686 PMC10161726

[9] Philippot L. , Griffiths B. S. , and Langenheder S. , Microbial Community Resilience Across Ecosystems and Multiple Disturbances, Microbiology and Molecular Biology Reviews. (2021) 85, no. 2, e00026-20, 10.1128/mmbr.00026-20, 33789927. 33789927 PMC8139522

[10] Ferone M. , Gowen A. , Fanning S. , and Scannell A. G. M. , Microbial Detection and Identification Methods: Bench Top Assays to Omics Approaches, Comprehensive Reviews in Food Science and Food Safety. (2020) 19, no. 6, 3106–3129, 10.1111/1541-4337.12618, 33337061. 33337061

[11] Idowu E. , Microbial Ecology: Understanding the Dynamics of Microbial Communities and Their Impact on Ecosystems, preprints, 2024 10.20944/preprints202412.0527.v1.

[12] Liu W. , Tang Y. , Zhang J. , Bai J. , Zhu Y. , Zhu L. , Zhao Y. , Daglia M. , Xiao X. , and He Y. , Microbial Interactions in Food Fermentation: Interactions, Analysis Strategies, and Quality Enhancement, Foods. (2025) 14, no. 14, 10.3390/foods14142515, 40724333. PMC12294674 40724333

[13] Trivedi P. , Leach J. E. , Tringe S. G. , Sa T. , and Singh B. K. , Plant–Microbiome Interactions: From Community Assembly to Plant Health, Nature Reviews Microbiology. (2020) 18, no. 11, 607–621, 10.1038/s41579-020-0412-1. 32788714

[14] Valiathan S. and Radhakrishnan P. , Applications of Biotechnological Approaches in the Food Industry, Novel And Alternative Methods In Food Processing, 2023, 1st edition, Apple Academic Press, 177–204, 10.1201/9781003328605-10.

[15] Sawant S. S. , Park H. Y. , Sim E. Y. , Kim H. S. , and Choi H. S. , Microbial Fermentation in Food: Impact on Functional Properties and Nutritional Enhancement—A Review of Recent Developments, Fermentation. (2025) 11, no. 1, 10.3390/fermentation11010015.

[16] Gänzle M. G. , Monnin L. , Zheng J. , Zhang L. , Coton M. , Sicard D. , and Walter J. , Starter Culture Development and Innovation for Novel Fermented Foods, Annual Review of Food Science and Technology. (2023) 15, no. 1, 211–239, 10.1146/annurev-food-072023-034207. 38052450

[17] Obayomi O. V. , Olaniran A. F. , and Owa S. O. , Unveiling the Role of Functional Foods With Emphasis on Prebiotics and Probiotics in Human Health: A Review, Journal of Functional Foods. (2024) 119, 106337, 10.1016/j.jff.2024.106337.

[18] Rameez K. M. , Santhoshkumar P. , Yoha K. S. , and Moses J. A. , Biopreservation of Food Using Probiotics: Approaches and Challenges, Current Research in Nutrition and Food Science Journal. (2024) 12, no. 2, 539–560, 10.12944/CRNFSJ.12.2.5.

[19] Arun K. , Anoopkumar A. N. , Sindhu R. , Binod P. , Aneesh E. M. , Madhavan A. , and Awasthi M. K. , Synthetic Biology for Sustainable Food Ingredients Production: Recent Trends, Systems Microbiology and Biomanufacturing. (2023) 3, no. 1, 137–149, 10.1007/s43393-022-00150-3.

[20] Lisboa H. M. , Pasquali M. B. , dos Anjos A. I. , Sarinho A. M. , de Melo E. D. , Andrade R. , Batista L. , Lima J. , Diniz Y. , and Barros A. , Innovative and Sustainable Food Preservation Techniques: Enhancing Food Quality, Safety, and Environmental Sustainability, Sustainability. (2024) 16, no. 18, 10.3390/su16188223.

[21] Widder S. , Allen R. J. , Pfeiffer T. , Curtis T. P. , Wiuf C. , Sloan W. T. , Cordero O. X. , Brown S. P. , Momeni B. , Shou W. , Kettle H. , Flint H. J. , Haas A. F. , Laroche B. , Kreft J. U. , Rainey P. B. , Freilich S. , Schuster S. , Milferstedt K. , van der Meer J. R. , Groβkopf T. , Huisman J. , Free A. , Picioreanu C. , Quince C. , Klapper I. , Labarthe S. , Smets B. F. , Wang H. , Isaac Newton Institute Fellows , and Soyer O. S. , Challenges in Microbial Ecology: Building Predictive Understanding of Community Function and Dynamics, ISME Journal. (2016) 10, no. 11, 2557–2568, 10.1038/ismej.2016.45, 27022995. 27022995 PMC5113837

[22] Alum E. A. , Urom S. , and Ben C. M. A. , Microbiological Contamination of Food: The Mechanisms, Impacts and Prevention, International Journal of Scientific Research and Technology. (2016) 5, no. 3, 65–78.

[23] Nerín C. , Aznar M. , and Carrizo D. , Food Contamination During Food Process, Trends In Food Science & Technology. (2016) 48, 63–68, 10.1016/j.tifs.2015.12.004.

[24] Iwu C. D. and Okoh A. I. , Preharvest Transmission Routes of Fresh Produce Associated Bacterial Pathogens With Outbreak Potentials: A Review, International Journal Of Environmental Research And Public Health. (2019) 16, no. 22, 10.3390/ijerph16224407, 31717976. PMC6888529 31717976

[25] Simothy L. , Mahomoodally F. , Neetoo H. , and Department of Agricultural and Food Sciences, Faculty of Agriculture, University of Mauritius, Réduit, Moka, 80837, Mauritius , A Study on the Potential of Ants to Act as Vectors of Foodborne Pathogens, AIMS Microbiology. (2018) 4, no. 2, 319–333, 10.3934/microbiol.2018.2.319, 31294218. 31294218 PMC6604928

[26] Shahanaz E. , Zwally K. M. , Powers C. , Lyons B. , Kaufman P. , Athrey G. , and Taylor T. M. , Flies as Vectors of Foodborne Pathogens Through Food Animal Production: Factors Affecting Pathogen and Antimicrobial Resistance Transmission, Journal of Food Protection. (2025) 88, no. 7, 100537, 10.1016/j.jfp.2025.100537, 40348086. 40348086

[27] Abebe G. M. , The Role of Bacterial Biofilm in Antibiotic Resistance and Food Contamination, International Journal Of Microbiology. (2020) 2020, no. 1, 1705814, 10.1155/2020/1705814. 32908520 PMC7468660

[28] Eng S.-K. , Pusparajah P. , Ab Mutalib N. S. , Ser H. L. , Chan K. G. , and Lee L. H. , Salmonella: A Review on Pathogenesis, Epidemiology and Antibiotic Resistance, Frontiers In Life Science. (2015) 8, no. 3, 284–293, 10.1080/21553769.2015.1051243.

[29] Wagenaar J. A. , French N. P. , and Havelaar A. H. , Preventing Campylobacter at the Source: Why Is It so Difficult?, Clinical Infectious Diseases. (2013) 57, no. 11, 1600–1606, 10.1093/cid/cit555, 24014733. 24014733

[30] Loor-Giler A. , Robayo-Chico M. , Puga-Torres B. , Hernandez-Alomia F. , Santander-Parra S. , Piantino Ferreira A. , Muslin C. , and Nuñez L. , Escherichia coli O157: H7, a Common Contaminant of Raw Milk From Ecuador: Isolation and Molecular Identification, Foods. (2025) 14, no. 3, 10.3390/foods14030410, 39942004. PMC11816838 39942004

[31] Onyeka L. O. , Adesiyun A. A. , Ismail A. , Allam M. , Keddy K. H. , and Thompson P. N. , Evidence for Horizontal Transmission and Recirculation of Shiga Toxin-Producing Escherichia coli in the Beef Production Chain in South Africa Using Whole Genome Sequencing, Pathogens. (2024) 13, no. 9, 10.3390/pathogens13090732, 39338923. PMC11434950 39338923

[32] Buchanan R. L. , Gorris L. G. M. , Hayman M. M. , Jackson T. C. , and Whiting R. C. , A Review of Listeria Monocytogenes: An Update on Outbreaks, Virulence, Dose-Response, Ecology, and Risk Assessments, Food Control. (2017) 75, 1–13, 10.1016/j.foodcont.2016.12.016.

[33] Vohra R. , Barash J. R. , Karmarkar E. N. , Koch-Kumar S. , Sanchez N. , Gore M. , Michel K. , Rangel M. , Armstrong E. , Pimentel L. , Kraushaar V. , Kimura A. , Stainken C. , Nat A. , Nat R. S. , Cherukupalli S. , Schneider D. , Vugia D. J. , Solis T. , Zweifler J. , Huntington S. , Prado J. , Luchini D. , and al Saghbini S. , Foodborne Botulism Outbreak After Consumption of Home-Canned Cactus (Nopales) - Fresno County, California, June 2024, Morbidity and Mortality Weekly Report. (2025) 74, no. 24, 408–413, 10.15585/mmwr.mm7424a1, 40608564. 40608564

[34] Agyirifo D. S. , Mensah T. A. , Senya A. S. Y. , Hounkpe A. , Dornyoh C. D. , and Otwe E. P. , Dynamics of Antimicrobial Resistance and Virulence of Staphylococcal Species Isolated From Foods Traded in the Cape Coast Metropolitan and Elmina Municipality of Ghana, Heliyon. (2023) 9, no. 11, e21584, 10.1016/j.heliyon.2023.e21584, 38027608. 38027608 PMC10663863

[35] Taddese D. , Abdurahaman M. , Debelo M. , Shumi E. , Urgessa G. , Kefyalew D. , Melaku M. , Kebeta T. , and Abafaji G. , Antimicrobial Resistance Characterization of Staphylococcus aureus From Different Animal Food Origins in Jimma, South Western Ethiopia, Discover Food. (2025) 5, no. 1, 10.1007/s44187-025-00300-1.

[36] Saba C. K. S. , Antwi M. V. , and Adzitey F. , Prevalence of Bacillus cereus in Ready-to-Eat Boiled and Fried Rice in the Tamale Metropolis of Ghana, Journal of Food Safety and Hygiene. (2019) 5, no. 1, 19–23.

[37] Green E. and Ogofure A. G. , Toxigenic and Antibiotic-Resistant Bacillus cereus in Raw Cow Milk From Eastern Cape, South Africa: A Potential Public Health Threat, Microorganisms. (2025) 13, no. 10, 10.3390/microorganisms13102253. PMC12566274 41156713

[38] Fokas R. , Anastopoulou Z. , Koukouvini K. A. , Dimitrakopoulou M. E. , Kotsiri Z. , Chorti-Tripsa E. , Kotsalou C. , Tzimotoudis D. , and Vantarakis A. , Long-Term Surveillance of Food Products of Diverse Origins: A Five-Year Survey of Hepatitis A and Norovirus in Greece, 2019–2024, Pathogens. (2025) 14, no. 2, 10.3390/pathogens14020135, 40005512. PMC11857987 40005512

[39] Zhu S. , Grant C. , Pan C. Y. , Adcock B. , Kao A. , Stous S. , Yee L. , Springfield O. , Poranski M. , Kennar A. , Beatty M. , Shah S. , Watson H. , Buonomo H. , Manlutac A. L. M. , Lima H. , Mendez D. , Clark B. , Pulido M. , Bakshi M. , Contreras C. , Green N. , Burleson T. , Forester J. , Klish S. W. , Feaster M. , Taylor E. V. , Balanji N. , Kho V. , Hatada A. , Wright C. , Morales C. , Abbott M. , Burditt F. R. , Elliot E. , Jones J. L. , Kinsey M. , Lombardi M. , Phelps K. , Woods J. W. , Kimura A. , and Lamba K. , Concurrent Norovirus Outbreaks Associated With Consumption of Oysters Harvested in Mexico—California, December 2023–January 2024, Morbidity and Mortality Weekly Report. (2025) 74, no. 13, 222–226, 10.15585/mmwr.mm7413a2, 40244935. 40244935 PMC12005483

[40] Mazaheri T. , Cervantes-Huamán B. R. H. , Bermúdez-Capdevila M. , Ripolles-Avila C. , and Rodríguez-Jerez J. J. , Listeria monocytogenes Biofilms in the Food Industry: Is the Current Hygiene Program Sufficient to Combat the Persistence of the Pathogen?, Microorganisms. (2021) 9, no. 1, 10.3390/microorganisms9010181, 33467747. PMC7830665 33467747

[41] Liu Y. , Zhu L. , Dong P. , Liang R. , Mao Y. , Yang X. , Zhang Y. , and Luo X. , Acid Tolerance Response of Listeria monocytogenes in Various External Phs With Different Concentrations of Lactic Acid, Foodborne Pathogens and Disease. (2020) 17, no. 4, 253–261, 10.1089/fpd.2019.2730, 31738578. 31738578

[42] Russell L. , Whyte P. , Zintl A. , Gordon S. V. , Markey B. , de Waal T. , Nolan S. , O′Flaherty V. , Abram F. , Richards K. , and Fenton O. , The Survival of *Salmonella* Senftenberg, *Escherichia coli* O157:H7, *Listeria monocytogenes*, *Enterococcus faecalis* and *Clostridium sporogenes* in Sandy and Clay Loam Textured Soils When Applied in Bovine Slurry or Unpasteurised Digestate and the Run-Off Rate for a Test Bacterium, *Listeria innocua*, When Applied to Grass in Slurry and Digestate, Frontiers in Sustainable Food Systems. (2022) 6, 806920, 10.3389/fsufs.2022.806920.

[43] Schoder D. , Guldimann C. , and Märtlbauer E. , Asymptomatic Carriage of Listeria monocytogenes by Animals and Humans and Its Impact on the Food Chain, Foods. (2022) 11, no. 21, 10.3390/foods11213472, 36360084. PMC9654558 36360084

[44] Gölz G. , Kittler S. , Malakauskas M. , and Alter T. , Survival of Campylobacter in the Food Chain and the Environment, Current Clinical Microbiology Reports. (2018) 5, no. 2, 126–134, 10.1007/s40588-018-0092-z.

[45] Bai X. , Nakatsu C. H. , and Bhunia A. K. , Bacterial Biofilms and Their Implications in Pathogenesis and Food Safety, Foods. (2021) 10, no. 9, 10.3390/foods10092117. PMC8472614 34574227

[46] Mohammadi B. , Reyes M. E. P. , and Smith S. A. , Survival, Growth, and Toxin Production of Bacillus cereus During Cooking and Storage of Fresh Rice Noodles, Journal of Food Protection. (2024) 87, no. 3, 100239, 10.1016/j.jfp.2024.100239, 38325555. 38325555

[47] Osek J. , Lachtara B. , and Wieczorek K. , Listeria monocytogenes–How This Pathogen Survives in Food-Production Environments?, Frontiers In Microbiology. (2022) 13, 866462, 10.3389/fmicb.2022.866462, 35558128. 35558128 PMC9087598

[48] Sharma D. , Pathogen Responses to Environmental Stressors in Pre-Harvest and Post-Harvest Stages of Produce Production, 2025, Michigan State University.

[49] Hellberg R. S. and Chu E. , Effects of Climate Change on the Persistence and Dispersal of Foodborne Bacterial Pathogens in the Outdoor Environment: A Review, Critical Reviews In Microbiology. (2016) 42, no. 4, 548–572, 10.3109/1040841X.2014.972335, 25612827. 25612827

[50] Legein M. , Smets W. , Vandenheuvel D. , Eilers T. , Muyshondt B. , Prinsen E. , Samson R. , and Lebeer S. , Modes of Action of Microbial Biocontrol in the Phyllosphere, Frontiers in Microbiology. (2020) 11, 10.3389/fmicb.2020.01619, 32760378. PMC7372246 32760378

[51] Mohanram S. and Kumar P. , Rhizosphere Microbiome: Revisiting the Synergy of Plant-Microbe Interactions, Annals of Microbiology. (2019) 69, no. 4, 307–320, 10.1007/s13213-019-01448-9.

[52] Truong H. N. , Garmyn D. , Gal L. , Fournier C. , Sevellec Y. , Jeandroz S. , and Piveteau P. , Plants as a Realized Niche for Listeria monocytogenes, Microbiologyopen. (2021) 10, no. 6, e1255, 10.1002/mbo3.1255, 34964288. 34964288 PMC8710918

[53] El-Ramady H. R. , Domokos-Szabolcsy É. , Abdalla N. A. , Taha H. S. , and Fári M. , Postharvest Management of Fruits and Vegetables Storage, Sustainable Agriculture Reviews:. (2015) 15, 65–152, 10.1007/978-3-319-09132-7_2.

[54] Liu X. , Yao H. , Zhao X. , and Ge C. , Biofilm Formation and Control of Foodborne Pathogenic Bacteria, Molecules. (2023) 28, no. 6, 10.3390/molecules28062432, 36985403. PMC10058477 36985403

[55] Fatima U. and Senthil-Kumar M. , Plant and Pathogen Nutrient Acquisition Strategies, Frontiers In Plant Science. (2015) 6, 10.3389/fpls.2015.00750. PMC4585253 26442063

[56] Weiss J. , Loeffler M. , and Terjung N. , The Antimicrobial Paradox: Why Preservatives Lose Activity in Foods, Current Opinion in Food Science. (2015) 4, 69–75, 10.1016/j.cofs.2015.05.008.

[57] Sharma R. , Garg P. , Kumar P. , Bhatia S. K. , and Kulshrestha S. , Microbial Fermentation and Its Role in Quality Improvement of Fermented Foods, Fermentation. (2020) 6, no. 4, 10.3390/fermentation6040106.

[58] Sionek B. , Szydłowska A. , Küçükgöz K. , and Kołożyn-Krajewska D. , Traditional and New Microorganisms in Lactic Acid Fermentation of Food, Fermentation. (2023) 9, no. 12, 10.3390/fermentation9121019.

[59] Li P. , Lin W. , Liu X. , Wang X. , and Luo L. , Environmental Factors Affecting Microbiota Dynamics During Traditional Solid-State Fermentation of Chinese Daqu Starter, Frontiers in Microbiology. (2016) 7, 10.3389/fmicb.2016.01237. PMC4972817 27540378

[60] Ji J. , Jiang X. , Song P. , Yang Q. , Sun M. , Dong Z. , Lu Y. , Dou S. , and Dong L. , Multi-Omics Insights Into Microbial Interactions and Fermented Food Quality, Microorganisms. (2025) 13, no. 12, 10.3390/microorganisms13122679, 41471883. PMC12734765 41471883

[61] Al-Kharousi Z. S. , Highlighting Lactic Acid Bacteria in Beverages: Diversity, Fermentation, Challenges, and Future Perspectives, Foods. (2025) 14, no. 12, 10.3390/foods14122043, 40565651. PMC12191484 40565651

[62] Olubukola O. , Azeta J. I. , and Obafemi Y. D. , Functional Use of Lactic Acid in Food Fermentation: Overview, Current Trends and Future Perspectives, Natural Product Research. (2024) 8, no. 11, 10.26538/tjnpr/v8i11.2.

[63] Walker G. M. and Stewart G. G. , Saccharomyces cerevisiae in the Production of Fermented Beverages, Beverages. (2016) 2, no. 4, 10.3390/beverages2040030.

[64] Pouris J. , Kolyva F. , Bratakou S. , Vogiatzi C. A. , Chaniotis D. , and Beloukas A. , The Role of Fungi in Food Production and Processing, Applied Sciences. (2024) 14, no. 12, 10.3390/app14125046.

[65] Lynch K. M. , Zannini E. , Wilkinson S. , Daenen L. , and Arendt E. K. , Physiology of Acetic Acid Bacteria and Their Role in Vinegar and Fermented Beverages, Comprehensive Reviews in Food Science and Food Safety. (2019) 18, no. 3, 587–625, 10.1111/1541-4337.12440, 33336918. 33336918

[66] Liu J. , Chan S. H. J. , Chen J. , Solem C. , and Jensen P. R. , Systems Biology–A Guide for Understanding and Developing Improved Strains of Lactic Acid Bacteria, Frontiers In Microbiology. (2019) 10, 10.3389/fmicb.2019.00876, 31114552. PMC6503107 31114552

[67] Abbaspour N. , Fermentation’s Pivotal Role in Shaping the Future of Plant-Based Foods: An Integrative Review of Fermentation Processes and Their Impact on Sensory and Health Benefits, Food Research. (2024) 4, no. 2, 100468, 10.1016/j.afres.2024.100468.

[68] Chen R. , Ren S. , Li S. , Zhou H. , Jia X. , Han D. , and Gao Z. , Synthetic Biology for the Food Industry: Advances and Challenges, Critical Reviews In Biotechnology. (2025) 45, no. 1, 23–47, 10.1080/07388551.2024.2340530. 38797660

[69] Rezac S. , Kok C. R. , Heermann M. , and Hutkins R. , Fermented Foods as a Dietary Source of Live Organisms, Frontiers In Microbiology. (2018) 9, 10.3389/fmicb.2018.01785, 30197628. PMC6117398 30197628

[70] Louw N. L. , Lele K. , Ye R. , Edwards C. B. , and Wolfe B. E. , Microbiome Assembly in Fermented Foods, Annual Review Of Microbiology. (2023) 77, no. 1, 381–402, 10.1146/annurev-micro-032521-041956. 37713453

[71] Tamang J. P. , Watanabe K. , and Holzapfel W. H. , Diversity of Microorganisms in Global Fermented Foods and Beverages, Frontiers In Microbiology. (2016) 7, 10.3389/fmicb.2016.00377. PMC4805592 27047484

[72] Valentino V. , Magliulo R. , Farsi D. , Cotter P. D. , O′Sullivan O. , Ercolini D. , and de Filippis F. , Fermented Foods, Their Microbiome and Its Potential in Boosting Human Health, Microbial Biotechnology. (2024) 17, no. 2, E14428, 10.1111/1751-7915.14428, 38393607. 38393607 PMC10886436

[73] Praveen M. and Brogi S. , Microbial Fermentation in Food and Beverage Industries: Innovations, Challenges, and Opportunities, Foods. (2025) 14, no. 1, 10.3390/foods14010114, 39796404. PMC11719914 39796404

[74] Dimidi E. , Cox S. R. , Rossi M. , and Whelan K. , Fermented Foods: Definitions and Characteristics, Impact on the Gut Microbiota and Effects on Gastrointestinal Health and Disease, Nutrients. (2019) 11, no. 8, 10.3390/nu11081806. PMC6723656 31387262

[75] De Filippis F. , Valentino V. , Yap M. , Cabrera-Rubio R. , Barcenilla C. , Carlino N. , Cobo-Díaz J. F. , Quijada N. M. , Calvete-Torre I. , Ruas-Madiedo P. , Sabater C. , Sequino G. , Pasolli E. , Wagner M. , Margolles A. , Segata N. , Álvarez-Ordóñez A. , Cotter P. D. , and Ercolini D. , Microbiome Mapping in Dairy Industry Reveals New Species and Genes for Probiotic and Bioprotective Activities, NPJ Biofilms And Microbiomes. (2024) 10, no. 1, 10.1038/s41522-024-00541-5, 39095404. PMC11297241 39095404

[76] Yang X. , Narvaez-Bravo C. , and Zhang P. , Driving Forces Shaping the Microbial Ecology in Meat Packing Plants, Frontiers In Microbiology. (2024) 14, 1333696, 10.3389/fmicb.2023.1333696, 38322759. 38322759 PMC10844536

[77] Hong X. , Chen J. , Liu L. , Wu H. , Tan H. , Xie G. , Xu Q. , Zou H. , Yu W. , Wang L. , and Qin N. , Metagenomic Sequencing Reveals the Relationship Between Microbiota Composition and Quality of Chinese Rice Wine, Scientific Reports. (2016) 6, no. 1, 26621, 10.1038/srep26621, 27241862. 27241862 PMC4886530

[78] Afshari R. , Pillidge C. J. , Read E. , Rochfort S. , Dias D. A. , Osborn A. M. , and Gill H. , New Insights Into Cheddar Cheese Microbiota-Metabolome Relationships Revealed by Integrative Analysis of Multi-Omics Data, Scientific Reports. (2020) 10, no. 1, 10.1038/s41598-020-59617-9, 32081987. PMC7035325 32081987

[79] Leech J. , Cabrera-Rubio R. , Walsh A. M. , Macori G. , Walsh C. J. , Barton W. , Finnegan L. , Crispie F. , O’Sullivan O. , Claesson M. J. , and Cotter P. D. , Fermented-Food Metagenomics Reveals Substrate-Associated Differences in Taxonomy and Health-Associated and Antibiotic Resistance Determinants, Msystems. (2020) 5, no. 6, 10.1128/mSystems.00522-20, 33172966. PMC7657593 33172966

[80] Elcheninov A. G. , Zayulina K. S. , Klyukina A. A. , Kremneva M. K. , Kublanov I. V. , and Kochetkova T. V. , Metagenomic Insights Into the Taxonomic and Functional Features of Traditional Fermented Milk Products From Russia, Microorganisms. (2024) 12, no. 1, 10.3390/microorganisms12010016, 38276185. PMC10819033 38276185

[81] Hwang B. K. , Choi H. L. , Choi S. H. , and Kim B. S. , Analysis of Microbiota Structure and Potential Functions Influencing Spoilage of Fresh Beef Meat, Frontiers In Microbiology. (2020) 11, 10.3389/fmicb.2020.01657, 32793151. PMC7387507 32793151

[82] Asmus A. , Gaire T. N. , Heimer K. M. , Belk K. E. , Singer R. S. , Johnson T. J. , and Noyes N. R. , Fresh Pork Microbiota Is Temporally Dynamic and Compositionally Diverse Across Meat, Contact Surfaces, and Processing Lines in a Pork Processing Facility, Applied And Environmental Microbiology. (2025) 91, no. 4, e00044-25, 10.1128/aem.00044-25, 40178255. 40178255 PMC12016530

[83] Yuan L. , Fan L. , Hou J. , Luo R. , Wang S. , Zhou W. , and Yang Z. , Metagenomic Analysis Reveals Microbial Community and Functional Capacity in Kombucha, Quality Assurance And Safety Of Crops & Foods. (2022) 14, no. 3, 1–8, 10.15586/qas.v14i3.1102.

[84] Yasir M. , Al-Zahrani I. A. , Bibi F. , Abd El Ghany M. , and Azhar E. I. , New Insights of Bacterial Communities in Fermented Vegetables From Shotgun Metagenomics and Identification of Antibiotic Resistance Genes and Probiotic Bacteria, Food Research International. (2022) 157, 111190, 10.1016/j.foodres.2022.111190. 35761518

[85] Syropoulou F. , Anagnostopoulos D. A. , Parlapani F. F. , Karamani E. , Stamatiou A. , Tzokas K. , Nychas G. J. E. , and Boziaris I. S. , Microbiota Succession of Whole and Filleted European Sea Bass (*Dicentrarchus labrax*) During Storage Under Aerobic and MAP Conditions via 16S Rrna Gene High-Throughput Sequencing Approach, Microorganisms. (2022) 10, no. 9, 10.3390/microorganisms10091870, 36144472. PMC9505548 36144472

[86] Yumnam H. , Hazarika P. , and Sharma I. , Metagenomic Insights Into Traditional Fermentation of Rice-Based Beverages Among Ethnic Tribes in Southern Assam, Northeast India, Northeast India. Frontiers In Microbiology. (2024) 15, 1410098, 10.3389/fmicb.2024.1410098. 39380672 PMC11459095

[87] Benucci G. M. N. , Wang X. , Zhang L. , Bonito G. , and Yu F. , Yeast and Lactic Acid Bacteria Dominate the Core Microbiome of Fermented ‘Hairy’ Tofu (Mao Tofu), Diversity. (2022) 14, no. 3, 10.3390/d14030207.

[88] Okpara M. O. , Microbial Enzymes and Their Applications in Food Industry: A Mini-Review, Advances In Enzyme Research. (2022) 10, no. 1, 23–47, 10.4236/aer.2022.101002.

[89] Kumar A. , Dhiman S. , Krishan B. , Samtiya M. , Kumari A. , Pathak N. , Kumari A. , Aluko R. E. , and Dhewa T. , Microbial Enzymes and Major Applications in the Food Industry: A Concise Review, Food Production, Processing And Nutrition. (2024) 6, no. 1, 10.1186/s43014-024-00261-5.

[90] Kumar D. , A Review of the Use of Microbial Amylase in Industry, Asian Journal Of Research In Social Sciences And Humanities. (2021) 11, no. 12, 57–61, 10.5958/2249-7315.2021.00321.X.

[91] Bock J. , Enzymes in Breadmaking, Improving and Tailoring Enzymes for Food Quality And Functionality, 2024, Elsevier, 217–239, 10.1016/B978-0-443-15437-9.00003-3.

[92] Saini R. , Saini H. S. , and Dahiya A. , Amylases: Characteristics and Industrial Applications, Journal of Pharmacognosy and Phytochemistry. (2017) 6, no. 4, 1865–1871.

[93] Ward O. , Proteases, Comprehensive biotechnology. (2019) .

[94] Li L. , Pei Y. , Cheng K. , Deng Y. , Dong X. , Fang R. , Chu B. , Wei P. , Chen Q. , and Xiao G. , Production and Evaluation of Enzyme-Modified Cheese Adding Protease or Lipase to Improve Quality Properties, Journal Of Bioscience And Bioengineering. (2023) 135, no. 5, 389–394, 10.1016/j.jbiosc.2023.02.006. 36922316

[95] Gagaoua M. , Dib A. L. , Lakhdara N. , Lamri M. , Botineştean C. , and Lorenzo J. M. , Artificial Meat Tenderization Using Plant Cysteine Proteases, Current Opinion In Food Science. (2021) 38, 177–188, 10.1016/j.cofs.2020.12.002.

[96] Sarmah N. , Revathi D. , Sheelu G. , Yamuna Rani K. , Sridhar S. , Mehtab V. , and Sumana C. , Recent Advances on Sources and Industrial Applications of Lipases, Biotechnology Progress. (2018) 34, no. 1, 5–28, 10.1002/btpr.2581, 29086509. 29086509

[97] Khan U. and Selamoglu Z. , Use of Enzymes in Dairy Industry: A Review of Current Progress, Archives Of Razi Institute. (2020) 75, no. 1, 131–136, 10.22092/ari.2019.126286.1341. 32292011 PMC8410156

[98] Keshavarz B. and Khalesi M. , Trichoderma reesei, a Superior Cellulase Source for Industrial Applications, Biofuels. (2016) 7, no. 6, 713–721, 10.1080/17597269.2016.1192444.

[99] De Souza T. S. and Kawaguti H. Y. , Cellulases, Hemicellulases, and Pectinases: Applications in the Food and Beverage Industry, Food And Bioprocess Technology. (2021) 14, no. 8, 1446–1477, 10.1007/s11947-021-02678-z.

[100] Haile S. and Ayele A. , Pectinase From Microorganisms and Its Industrial Applications, The Scientific World Journal. (2022) 2022, no. 1, 1881305, 10.1155/2022/1881305. 35311220 PMC8933074

[101] Sharma H. P. and Patel H. , Enzymatic Added Extraction and Clarification of Fruit Juices–A Review, Critical Reviews In Food Science and Nutrition. (2017) 57, no. 6, 1215–1227, 10.1080/10408398.2014.977434, 26731188. 26731188

[102] Mao S. , Jiang J. , Xiong K. , Chen Y. , Yao Y. , Liu L. , Liu H. , and Li X. , Enzyme Engineering: Performance Optimization, Novel Sources, and Applications in the Food Industry, Foods. (2024) 13, no. 23, 10.3390/foods13233846, 39682920. PMC11639928 39682920

[103] Rigoldi F. , Donini S. , Redaelli A. , Parisini E. , and Gautieri A. , Review: Engineering of Thermostable Enzymes for Industrial Applications, APL Bioengineering. (2018) 2, no. 1, 011501, 10.1063/1.4997367, 31069285. 31069285 PMC6481699

[104] Prayogo F. A. , Budiharjo A. , Kusumaningrum H. P. , Wijanarka W. , Suprihadi A. , and Nurhayati N. , Metagenomic Applications in Exploration and Development of Novel Enzymes From Nature: A Review, Journal of Genetic Engineering and Biotechnology. (2020) 18, no. 1, 10.1186/s43141-020-00043-9. PMC7403272 32749574

[105] Cui J. , Mai G. , Wang Z. , Liu Q. , Zhou Y. , Ma Y. , and Liu C. , Metagenomic Insights Into a Cellulose-Rich Niche Reveal Microbial Cooperation in Cellulose Degradation, Frontiers In Microbiology. (2019) 10, 10.3389/fmicb.2019.00618, 30984144. PMC6447707 30984144

[106] Zuo J. , Zhang J. , Ma H. , Zhang Y. , Li P. , Wu Y. , Tian P. , Fan Q. , Cao L. , Sun J. , and Gu S. , Site-Directed Mutagenesis Increased the Catalytic Activity and Stability of Oenococcus oeni *β*-Glucosidase: Characterization of Enzymatic Properties and Exploration of Mechanisms, International Journal of Molecular Sciences. (2025) 26, no. 9, 10.3390/ijms26093983, 40362224. PMC12072002 40362224

[107] Borchert E. , García-Moyano A. , Sanchez-Carrillo S. , Dahlgren T. G. , Slaby B. M. , Bjerga G. E. K. , Ferrer M. , Franzenburg S. , and Hentschel U. , Deciphering a Marine Bone-Degrading Microbiome Reveals a Complex Community Effort, mSystems. (2021) 6, no. 1, e01218-20, 10.1128/Msystems.01218-20. 33563781 PMC7883544

[108] García-Moyano A. , Diaz Y. , Navarro J. , Almendral D. , Puntervoll P. , Ferrer M. , and Bjerga G. E. K. , Two-Step Functional Screen on Multiple Proteinaceous Substrates Reveals Temperature-Robust Proteases With a Broad-Substrate Range, Applied Microbiology and Biotechnology. (2021) 105, no. 8, 3195–3209, 10.1007/s00253-021-11235-9, 33770243. 33770243 PMC8053189

[109] Motahar S. F. S. , Khatibi A. , Salami M. , Ariaeenejad S. , Emam-Djomeh Z. , Nedaei H. , Kavousi K. , Sheykhabdolahzadeh Mamaghani A. , and Salekdeh G. H. , A Novel Metagenome-Derived Thermostable and Poultry Feed Compatible *Α*-Amylase With Enhanced Biodegradation Properties, International Journal Of Biological Macromolecules. (2020) 164, 2124–2133, 10.1016/j.ijbiomac.2020.08.064, 32795571. 32795571

[110] Menshawy M. N. , Abdel-Hamid A. M. , el-Katatny M.’. H. , and Arafat H. H. , Maximizing Cellulase and Xylanase Production From Novel Bacillus pumilus Strain Isolated From Agricultural Waste Compost in Egypt and Optimizing Their Activities, Discover Applied Sciences. (2025) 7, no. 10, 10.1007/s42452-025-07058-2.

[111] Wan M. L. , Ling K. H. , el-Nezami H. , and Wang M. F. , Influence of Functional Food Components on Gut Health, Critical Reviews In Food Science And Nutrition. (2019) 59, no. 12, 1927–1936, 10.1080/10408398.2018.1433629. 29381385

[112] Martín R. and Langella P. , Emerging Health Concepts in the Probiotics Field: Streamlining the Definitions, Frontiers In Microbiology. (2019) 10, 10.3389/fmicb.2019.01047, 31164874. PMC6536656 31164874

[113] Vandenplas Y. , Huys G. , and Daube G. , Probiotics: An Update, Jornal De Pediatria. (2015) 91, no. 1, 6–21, 10.1016/j.jped.2014.08.005. 25458874

[114] Soares M. B. , Almada C. N. , Pereira E. P. R. , Ferreira B. M. , Balthazar C. F. , Khorshidian N. , Rocha R. S. , Xavier-Santos D. , Cruz A. G. , Ranadheera C. S. , Mortazavian A. M. , Gómez-Zavaglia A. , Martinez R. C. R. , and Sant’Ana A. S. , Review - Sporeforming Probiotic Bacteria: Characteristics, Health Benefits, and Technological Aspects for Their Applications in Foods and Beverages, Trends In Food Science & Technology. (2023) 138, 453–469, 10.1016/j.tifs.2023.06.029.

[115] Baliyan N. , Kumari M. , Kumari P. , Dindhoria K. , Mukhia S. , Kumar S. , Gupta M. , and Kumar R. , Probiotics in Fermented Products and Supplements, Current Developments In Biotechnology And Bioengineering, 2022, Elsevier, 73–107, 10.1016/B978-0-12-823506-5.00014-X.

[116] Khaneghah A. M. , Abhari K. , Eş I. , Soares M. B. , Oliveira R. B. , Hosseini H. , Rezaei M. , Balthazar C. F. , Silva R. , Cruz A. G. , and Ranadheera C. S. , Interactions Between Probiotics and Pathogenic Microorganisms in Hosts and Foods: A Review, Trends In Food Science & Technology. (2020) 95, 205–218, 10.1016/j.tifs.2019.11.022.

[117] Monika K. , Malik T. , Gehlot R. , Rekha K. , Kumari A. , Sindhu R. , and Rohilla P. , Antimicrobial Property of Probiotics, Environment Conservation Journal. (2021) 22, 33–48, 10.36953/ECJ.2021.SE.2204.

[118] La Fata G. , Weber P. , and Mohajeri M. H. , Probiotics and the Gut Immune System: Indirect Regulation, Probiotics And Antimicrobial Proteins. (2018) 10, no. 1, 11–21, 10.1007/s12602-017-9322-6, 28861741. 28861741 PMC5801397

[119] Tang C. and Lu Z. , Health Promoting Activities of Probiotics, Journal Of Food Biochemistry. (2019) 43, no. 8, e12944, 10.1111/jfbc.12944, 31368544. 31368544

[120] Ricke S. C. , Lee S. I. , Kim S. A. , Park S. H. , and Shi Z. , Prebiotics and the Poultry Gastrointestinal Tract Microbiome, Poultry Science. (2020) 99, no. 2, 670–677, 10.1016/j.psj.2019.12.018, 32029153. PMC7587714 32029153

[121] Martyniak A. , Medyńska-Przęczek A. , Wędrychowicz A. , Skoczeń S. , and Tomasik P. J. , Prebiotics, Probiotics, Synbiotics, Paraprobiotics and Postbiotic Compounds in IBD, Biomolecules. (2021) 11, no. 12, 10.3390/biom11121903, 34944546. PMC8699341 34944546

[122] Fernández J. , Redondo-Blanco S. , Gutiérrez-del-Río I. , Miguélez E. M. , Villar C. J. , and Lombó F. , Colon Microbiota Fermentation of Dietary Prebiotics Towards Short-Chain Fatty Acids and Their Roles as Anti-Inflammatory and Antitumour Agents: A Review, Journal Of Functional Foods. (2016) 25, 511–522, 10.1016/j.jff.2016.06.032.

[123] Malos I. G. , Pasarin D. , Ghizdareanu A. I. , and Frunzareanu B. , A Promising Approach for the Food Industry: Enhancing Probiotic Viability Through Microencapsulated Synbiotics, Microorganisms. (2025) 13, no. 2, 10.3390/microorganisms13020336, 40005703. PMC11858381 40005703

[124] Dutta S. , Chatterjee N. , Gallina N. L. F. , Kar S. , Koley H. , Nanda P. K. , Biswas O. , Das A. K. , Biswas S. , Bhunia A. K. , and Dhar P. , Diet, Microbiome, and Probiotics Establish a Crucial Link in Vaccine Efficacy, Critical Reviews In Microbiology. (2025) 51, 1–26, 10.1080/1040841X.2025.2480230. 40110742

[125] Al-Fakhrany O. M. and Elekhnawy E. , Next-Generation Probiotics: The Upcoming Biotherapeutics, Molecular Biology Reports. (2024) 51, no. 1, 10.1007/s11033-024-09398-5, 38619680. PMC11018693 38619680

[126] Chauhan K. and Rao A. , Clean-Label Alternatives for Food Preservation: An Emerging Trend, Heliyon. (2024) 10, no. 16, e35815, 10.1016/j.heliyon.2024.e35815, 39247286. 39247286 PMC11379619

[127] Varsha K. K. and Nampoothiri K. M. , Appraisal of Lactic Acid Bacteria as Protective Cultures, Food Control. (2016) 69, 61–64, 10.1016/j.foodcont.2016.04.032.

[128] Ibrahim S. A. , Ayivi R. D. , Zimmerman T. , Siddiqui S. A. , Altemimi A. B. , Fidan H. , Esatbeyoglu T. , and Bakhshayesh R. V. , Lactic Acid Bacteria as Antimicrobial Agents: Food Safety and Microbial Food Spoilage Prevention, Foods. (2021) 10, no. 12, 10.3390/foods10123131, 34945682. PMC8701396 34945682

[129] Ben Said L. , Gaudreau H. , Dallaire L. , Tessier M. , and Fliss I. , Bioprotective Culture: A New Generation of Food Additives for the Preservation of Food Quality and Safety, Industrial Biotechnology. (2019) 15, no. 3, 138–147, 10.1089/ind.2019.29175.lbs.

[130] Todorov S. D. , Popov I. , Weeks R. , and Chikindas M. L. , Use of Bacteriocins and Bacteriocinogenic Beneficial Organisms in Food Products: Benefits, Challenges, Concerns, Foods. (2022) 11, no. 19, 10.3390/foods11193145, 36230222. PMC9563261 36230222

[131] Rebuffat S. , Ribosomally Synthesized Peptides, Foreground Players in Microbial Interactions: Recent Developments and Unanswered Questions, Natural Product Reports. (2022) 39, no. 2, 273–310, 10.1039/D1NP00052G, 34755755. 34755755

[132] Bangar S. P. , Chaudhary V. , Singh T. P. , and Özogul F. , Retrospecting the Concept and Industrial Significance of LAB Bacteriocins, Food Bioscience. (2022) 46, 101607, 10.1016/j.fbio.2022.101607.

[133] Parada Fabián J. C. , Álvarez Contreras A. K. , Natividad Bonifacio I. , Hernández Robles M. F. , Vázquez Quiñones C. R. , Quiñones Ramírez E. I. , and Vázquez Salinas C. , Toward Safer and Sustainable Food Preservation: A Comprehensive Review of Bacteriocins in the Food Industry, Bioscience Reports. (2025) 45, no. 4, BSR20241594, 10.1042/BSR20241594. 40259615 PMC12203937

[134] Sugrue I. , Ross R. P. , and Hill C. , Bacteriocin Diversity, Function, Discovery and Application as Antimicrobials, Nature Reviews Microbiology. (2024) 22, no. 9, 556–571, 10.1038/s41579-024-01045-x, 38730101. 38730101 PMC7616364

[135] Charest A. M. , Reed E. , Bozorgzadeh S. , Hernandez L. , Getsey N. V. , Smith L. , Galperina A. , Beauregard H. E. , Charest H. A. , Mitchell M. , and Riley M. A. , Nisin Inhibition of Gram-Negative Bacteria, Microorganisms. (2024) 12, no. 6, 10.3390/microorganisms12061230, 38930612. PMC11205666 38930612

[136] Toussaint B. , Munoz-Pineiro A. , and Pirnay J. , Overview and Outlook of Phage Therapy and Phage Biocontrol, 2024, Publications Office of the European Union: Luxembourg.

[137] Wu Q. , Li L. , Xiang P. , Zhang T. , Peng L. , Zou L. , and Li Q. , Phages in Fermented Foods: Interactions and Applications, Fermentation. (2023) 9, no. 3, 10.3390/fermentation9030201.

[138] Jaglan A. B. , Anand T. , Verma R. , Vashisth M. , Virmani N. , Bera B. C. , Vaid R. K. , and Tripathi B. N. , Tracking the Phage Trends: A Comprehensive Review of Applications in Therapy and Food Production, Frontiers in Microbiology. (2022) 13, 993990, 10.3389/fmicb.2022.993990, 36504807. 36504807 PMC9730251

[139] Ribes S. , Fuentes A. , Talens P. , and Barat J. M. , Prevention of Fungal Spoilage in Food Products Using Natural Compounds: A Review, Critical Reviews In Food Science And Nutrition. (2018) 58, no. 12, 2002–2016, 10.1080/10408398.2017.1295017, 28394635. 28394635

[140] Liu A. , Xu R. , Zhang S. , Wang Y. , Hu B. , Ao X. , Li Q. , Li J. , Hu K. , Yang Y. , and Liu S. , Antifungal Mechanisms and Application of Lactic Acid Bacteria in Bakery Products: A Review, Frontiers in Microbiology. (2022) 13, 924398, 10.3389/fmicb.2022.924398, 35783382. 35783382 PMC9244174

[141] Aladhadh M. , A Review of Modern Methods for the Detection of Foodborne Pathogens, Microorganisms. (2023) 11, no. 5, 10.3390/microorganisms11051111, 37317085. PMC10221273 37317085

[142] Gomes E. , Araújo D. , Nogueira T. , Oliveira R. , Silva S. , Oliveira L. V. N. , Azevedo N. F. , Almeida C. , and Castro J. , Advances in Whole Genome Sequencing for Foodborne Pathogens: Implications for Clinical Infectious Disease Surveillance and Public Health, Frontiers in Cellular and Infection Microbiology. (2025) 15, 1593219, 10.3389/fcimb.2025.1593219, 40357405. 40357405 PMC12066639

[143] Ahmad S. , Lohiya S. , Taksande A. , Meshram R. J. , Varma A. , and Vagha K. , A Comprehensive Review of Innovative Paradigms in Microbial Detection and Antimicrobial Resistance: Beyond Traditional Cultural Methods, Cureus. (2024) 16, no. 6, 10.7759/cureus.61476. PMC11216122 38952583

[144] Imanian B. , Donaghy J. , Jackson T. , Gummalla S. , Ganesan B. , Baker R. C. , Henderson M. , Butler E. K. , Hong Y. , Ring B. , Thorp C. , Khaksar R. , Samadpour M. , Lawless K. A. , MacLaren-Lee I. , Carleton H. A. , Tian R. , Zhang W. , and Wan J. , The Power, Potential, Benefits, and Challenges of Implementing High-Throughput Sequencing in Food Safety Systems, NPJ Science of Food. (2022) 6, no. 1, 10.1038/s41538-022-00150-6, 35974024. PMC9381742 35974024

[145] Yap M. , Ercolini D. , Álvarez-Ordóñez A. , O′Toole P. W. , O′Sullivan O. , and Cotter P. D. , Next-Generation Food Research: Use of Meta-Omic Approaches for Characterizing Microbial Communities Along the Food Chain, Annual Review of Food Science and Technology. (2022) 13, no. 1, 361–384, 10.1146/annurev-food-052720-010751, 34678075. 34678075

[146] Wang J. , den Bakker H. C. , and Denes T. G. , Food-Derived Extracellular Vesicles: An Emerging Intervention Strategy for Inflammatory Bowel Disease, Critical Reviews in Food Science and Nutrition. (2025) 1–22, 10.1080/10408398.2025.2603666, 41431254. 41431254

[147] Chandross-Cohen T. , Chung T. , Watson S. C. , Rolon M. L. , and Kovac J. , Precision Food Safety: Advances in Omics-Based Surveillance for Proactive Detection and Management of Foodborne Pathogens, Trends in Food Science & Technology. (2025) 163, 105186, 10.1016/j.tifs.2025.105186.

[148] Lamas A. , Regal P. , Vázquez B. , Miranda J. M. , Franco C. M. , and Cepeda A. , Transcriptomics: A Powerful Tool to Evaluate the Behavior of Foodborne Pathogens in the Food Production Chain, Food Research International. (2019) 125, 108543, 10.1016/j.foodres.2019.108543, 31554082. 31554082

[149] Sidira M. , Smaoui S. , and Varzakas T. , Recent Proteomics, Metabolomics and Lipidomics Approaches in Meat Safety, Processing and Quality Analysis, Applied Sciences. (2024) 14, no. 12, 10.3390/app14125147.

[150] Hayoglu I. , Guclu S. , and Hayoglu B. , Metabolomics and Proteomics in Foods. International Journal Of Current Naturalscience And Advanced, Phytochemistry. (2024) 4, no. 1.

[151] Boutsika A. , Michailidis M. , Ganopoulou M. , Dalakouras A. , Skodra C. , Xanthopoulou A. , Stamatakis G. , Samiotaki M. , Tanou G. , Moysiadis T. , Angelis L. , Bazakos C. , Molassiotis A. , Nianiou-Obeidat I. , Mellidou I. , and Ganopoulos I. , A Wide Foodomics Approach Coupled With Metagenomics Elucidates the Environmental Signature of Potatoes, Iscience. (2023) 26, no. 1, 105917, 10.1016/j.isci.2022.105917, 36691616. 36691616 PMC9860355

[152] Liu S. , Li D. , Zhao X. , Qin Z. , Zeng W. , and Zhou J. , Production of Food Flavor and Color by Synthetic Biology, Current Opinion in Food Science. (2024) 57, 101168, 10.1016/j.cofs.2024.101168.

[153] Pham V. D. , Simpson D. J. , and Gänzle M. G. , Strain-Level Identification of Bacteria Persisting in Food and in Food Processing Facilities: When Do Two Isolates Represent the Same Strain and Which Tools Identify a Strain?, Current Opinion in Food Science. (2025) 61, 101245, 10.1016/j.cofs.2024.101245.

[154] WHO , Whole Genome Sequencing as a Tool to Strengthen Foodborne Disease Surveillance and Response. Module 3: Whole Genome Sequencing in Foodborne Disease Routine Surveillance, 2023, https://www.who.int/publications/i/item/9789240021266.

[155] Dong K. , Song D. , Li S. , Wang X. , Dai L. , Pei X. , Yang X. , and Jiang Y. , Significance of Whole-Genome Sequencing for the Traceability of Foodborne Pathogens: During the Processing of Meat and Dairy Products, Foods. (2025) 14, no. 8, 10.3390/foods14081410, 40282811. PMC12026735 40282811

[156] Ray L. C. , Griffin P. M. , Wymore K. , Wilson E. , Hurd S. , LaClair B. , Wozny S. , Eikmeier D. , Nicholson C. , Burzlaff K. , Hatch J. , Fankhauser M. , Kubota K. , Huang J. Y. , Geissler A. , Payne D. C. , and Tack D. M. , Changing Diagnostic Testing Practices for Foodborne Pathogens, Foodborne Diseases Active Surveillance Network, 2012–2019, Open Forum Infectious Diseases. (2022) 9, no. 8, ofac344, 10.1093/ofid/ofac344, 35928506. 35928506 PMC9345410

[157] Srinivas M. , O’Sullivan O. , Cotter P. D. , Sinderen D. , and Kenny J. G. , The Application of Metagenomics to Study Microbial Communities and Develop Desirable Traits in Fermented Foods, Foods. (2022) 11, no. 20, 10.3390/foods11203297, 37431045. PMC9601669 37431045

[158] Flörl L. , Meyer A. , and Bokulich N. A. , Exploring Sub-Species Variation in Food Microbiomes: A Roadmap to Reveal Hidden Diversity and Functional Potential, Applied and Environmental Microbiology. (2025) 91, no. 5, e00524-25, 10.1128/aem.00524-25. 40304520 PMC12093984

[159] Singh S. P. , Verma N. , Kumar D. , and Gupta S. , Computational Challenges in Metagenomic Data Analysis, Genomic Intelligence, 2024, CRC Press, 59–76.

[160] Parente E. , Zotta T. , and Ricciardi A. , FoodMicrobionet V4: A Large, Integrated, Open and Transparent Database for Food Bacterial Communities, International Journal of Food Microbiology. (2022) 372, 109696, 10.1016/j.ijfoodmicro.2022.109696, 35526357. 35526357

[161] Seneviratne C. J. , Suriyanarayanan T. , Widyarman A. S. , Lee L. S. , Lau M. , Ching J. , Delaney C. , and Ramage G. , Multi-Omics Tools for Studying Microbial Biofilms: Current Perspectives and Future Directions, Critical Reviews in Microbiology. (2020) 46, no. 6, 759–778, 10.1080/1040841X.2020.1828817, 33030973. 33030973

[162] Ferrocino I. , Rantsiou K. , McClure R. , Kostic T. , de Souza R. S. C. , Lange L. , FitzGerald J. , Kriaa A. , Cotter P. , Maguin E. , Schelkle B. , Schloter M. , Berg G. , Sessitsch A. , Cocolin L. , and The MicrobiomeSupport Consortium , The Need for an Integrated Multi-Omics Approach in Microbiome Science in the Food System, Comprehensive Reviews in Food Science and Food Safety. (2023) 22, no. 2, 1082–1103, 10.1111/1541-4337.13103, 36636774. 36636774

[163] Shi S. , Wang Z. , Shen L. , and Xiao H. , Synthetic Biology: A New Frontier in Food Production, Trends in Biotechnology. (2022) 40, no. 7, 781–803, 10.1016/j.tibtech.2022.01.002. 35120749

[164] Ramírez Rojas A. A. , Swidah R. , and Schindler D. , Microbes of Traditional Fermentation Processes as Synthetic Biology Chassis to Tackle Future Food Challenges, Frontiers in Bioengineering and Biotechnology. (2022) 10, 982975, 10.3389/fbioe.2022.982975, 36185425. 36185425 PMC9523148

[165] Lin L. , Bottom-Up Synthetic Ecology Study of Microbial Consortia to Enhance Lignocellulose Bioconversion, Biotechnology for Biofuels and Bioproducts. (2022) 15, no. 1, 10.1186/s13068-022-02113-1, 35418100. PMC8822760 35418100

[166] Berkhout M. , Zoetendal E. , Plugge C. , and Belzer C. , Use of Synthetic Communities to Study Microbial Ecology of the Gut, Microbiome Research Reports. (2022) 1, no. 1, 10.20517/mrr.2021.11, 38089065. PMC10714298 38089065

[167] Nikoloudaki O. , Aheto F. , di Cagno R. , and Gobbetti M. , Synthetic Microbial Communities: A Gateway to Understanding Resistance, Resilience, and Functionality in Spontaneously Fermented Food Microbiomes, Food Research International. (2024) 192, 114780, 10.1016/j.foodres.2024.114780, 39147468. 39147468

[168] Giaouris E. , Heir E. , Desvaux M. , Hébraud M. , Møretrø T. , Langsrud S. , Doulgeraki A. , Nychas G. J. , Kačániová M. , Czaczyk K. , Ölmez H. , and Simões M. , Intra-and Inter-Species Interactions Within Biofilms of Important Foodborne Bacterial Pathogens, Frontiers In Microbiology. (2015) 6, 10.3389/fmicb.2015.00841. PMC4542319 26347727

[169] Chen Q. , Zhang X. , Wang Q. , Yang J. , and Zhong Q. , The Mixed Biofilm Formed by Listeria monocytogenes and Other Bacteria: Formation, Interaction and Control Strategies, Critical Reviews in Food Science and Nutrition. (2024) 64, no. 24, 8570–8586, 10.1080/10408398.2023.2200861, 37070220. 37070220

[170] Pérez-Alvarado O. , Zepeda-Hernández A. , Garcia-Amezquita L. E. , Requena T. , Vinderola G. , and García-Cayuela T. , Role of Lactic Acid Bacteria and Yeasts in Sourdough Fermentation During Breadmaking: Evaluation of Postbiotic-Like Components and Health Benefits, Frontiers in Microbiology. (2022) 13, 969460, 10.3389/fmicb.2022.969460, 36187981. 36187981 PMC9524358

[171] Moye Z. D. , Woolston J. , and Sulakvelidze A. , Bacteriophage Applications for Food Production and Processing, Viruses. (2018) 10, no. 4, 10.3390/v10040205, 29671810. PMC5923499 29671810

[172] Leyva Salas M. , Mounier J. , Valence F. , Coton M. , Thierry A. , and Coton E. , Antifungal Microbial Agents for Food Biopreservation—A Review, Microorganisms. (2017) 5, no. 3, 10.3390/microorganisms5030037, 28698479. PMC5620628 28698479

[173] Mukherjee A. , Gómez-Sala B. , O′Connor E. M. , Kenny J. G. , and Cotter P. D. , Global Regulatory Frameworks for Fermented Foods: A Review, Frontiers In Nutrition. (2022) 9, 902642, 10.3389/fnut.2022.902642, 35719144. 35719144 PMC9198641

[174] Asquer A. and Krachkovskaya I. , Uncertainty, Institutions and Regulatory Responses to Emerging Technologies:CRISPRGene Editing in theUSand theEU(2012–2019), Regulation & Governance. (2021) 15, no. 4, 1111–1127, 10.1111/rego.12335.

[175] Tahyné Kovács Á. , The Development of GMO Regulatory Framework in the European Union: Enviromental Law Principles and Bioethical Challenges, Journal Of International Scientific Publications: Agriculture And Food. (2025) 13, no. 1, 139–157.

[176] Mathipa M. G. and Thantsha M. S. , Probiotic Engineering: Towards Development of Robust Probiotic Strains With Enhanced Functional Properties and for Targeted Control of Enteric Pathogens, Gut Pathogens. (2017) 9, no. 1, 10.1186/s13099-017-0178-9, 28491143. PMC5422995 28491143

